# Proteomic and transcriptomic analysis reveals an intracellular role for zona pellucida protein 2 in mouse morula-blastocyst transition

**DOI:** 10.1186/s12864-026-13487-z

**Published:** 2026-10-22

**Authors:** Thomas Nolte, Steffen Israel, Hannes C. A. Drexler, Georg Fuellen, Julina Wiese, Michele Boiani

**Affiliations:** 1 Department of Cell & Tissue Dynamics, Max Planck Institute for Molecular Biomedicine, Röntgenstrasse 20, 48149 Münster, Germany; 2 Department of Dermatology, University Hospital Münster, Von-Esmarch-Strasse 58, 48149 Münster, Germany; 3 Bioanalytical Mass Spectrometry Facility, Max Planck Institute for Molecular Biomedicine, Röntgenstrasse 20, 48149 Münster, Germany; 4 Institute for Biostatistics and Informatics in Medicine and Aging Research (IBIMA), Rostock University Medical Center, Ernst-Heydemann-Strasse 8, 18057 Rostock, Germany

**Keywords:** Animal model, Embryo development, Morpholino, Oocyte, Proteomics, RNA-seq, *Trim-Away*, Zona pellucida, ZP2

## Abstract

**Background:**

Since the first description in 1827, the zona pellucida (ZP) has been viewed as an extracellular structure surrounding mammalian oocytes, with three canonical roles: encasement of the oocyte in the follicle, sperm binding at fertilization, and physical protection of the early embryo. However, over the past 10 years, mass spectrometry analyses in this laboratory have repeatedly and consistently detected abundant ZP proteins inside mouse embryos. Therefore, this study investigated whether ZP2, the most abundant of the ZP proteins, might also serve non-canonical functions that operate from inside the embryo rather than from the outside.

**Results:**

Using mass spectrometry and monoclonal immunofluorescence, an intracellular fraction of ZP2 was found to persist throughout preimplantation mouse development, being among the most abundant proteins of the embryo. Intracellular ZP2 underwent an apparent increase at the 8-cell to morula stage rather than the monotonic decline expected of a passive maternal legacy. Basic colocalization analysis showed that part of this intracellular pool overlapped neither the cis-Golgi marker GM130 nor the endoplasmic reticulum marker Calreticulin, identifying a non-Golgi, non-ER intracellular ZP2 fraction. Consistent with this, ZP2 was susceptible to *Trim-Away* mediated depletion, which typically can act on a protein only if this is cytosolically accessible. Although ZP2 depletion stalled at approximately 50% due to the enormous abundance of ZP2, this partial knockdown delayed the morula-to-blastocyst transition and produced blastocysts with reduced trophectoderm and primitive endoderm and impaired postimplantation development in vitro and in vivo. These effects were specific to ZP2, as they did not occur when *Trim-Away* was redirected to an irrelevant target. Proteome and transcriptome analyses of ZP2 knockdown embryos returned overrepresented gene ontology terms related to cytoplasmic translation in the proteome, which was confirmed by an O-propargyl-puromycin incorporation assay; and to endoderm development and gastrulation in the transcriptome, which provide a coherent molecular correlate of the morula retardation phenotype.

**Conclusions:**

These results confer ZP2 with hitherto unsuspected intracellular roles during embryogenesis in the mouse model of human reproduction. ZP2 can therefore no longer be referred to as a solely extracellular-operating, oocyte-specific gene product.

**Supplementary Information:**

The online version contains supplementary material available at https://doi.org/10.1186/s12864-026-13487-z.

## Background

It has been nearly 200 years since Karl Ernst von Baer described the quintessential phenotypic structure of mammalian oocytes, the zona pellucida (ZP) [1]. We know from numerous subsequent studies that the ZP is a secreted glycoprotein matrix composed of 3–4 glycoproteins (ZP1, ZP2, ZP3, ZP4 in humans; ZP1, ZP2, ZP3 in mice) that are synthesized only during oogenesis and compartmentalized into the secretory pathway [2–5]. Once secreted and assembled around the oocyte, ZP proteins are tasked with encasing the oocyte in the ovarian follicle during oogenesis, mediating sperm-oocyte interaction and monospermic fertilization during conception, and preventing premature contact between the embryo and the oviductal epithelium during preimplantation development. These three functions all have in common that they are extracellular, originate in the oocyte, and are passively transmitted to the embryo. However, this model is probably incomplete. First, it does not explain why, in the original study that deleted the *Zp2* locus in mice [6], the embryos obtained from *Zp2*-null oocytes were developmentally impaired even if the problem caused by the mutation had been circumvented. Specifically, the mutant zona was thin and this caused polyspermy with consequent embryonic failure due to polyploidy. However, even when *Zp2*-null oocytes were fertilized monospermically in vitro (by lowering sperm concentration) and blastocysts were obtained, no live births were obtained after transfer to pseudopregnant females [6]. Apparently, this was due to compromised oocyte quality arising from disrupted oocyte–granulosa communication during folliculogenesis. However, this upstream role does not rule out the possibility that ZP2 may also be needed in embryos. Second, the model does not accommodate more recent hints that the synthesis of ZP proteins might take place also after oogenesis, as supported by the finding of *Zp* genes’ mRNAs bound to polyribosome of blastocyst-stage mouse embryos [7, 8]. Third, it was reported that the abundance of ZP2 was not declining during preimplantation development, as would be expected of a maternal protein synthesized only during oogenesis, but seemed to increase at the blastocyst stage (Supplementary Table S1 in [9]). Fourth and last, a burst of *Zp3* transcription was described in E4.5 epiblast (one of the cell lineages of blastocysts), following computational re-analysis of public scRNA-seq datasets [10]. Points 2, 3, and 4 do not align with the notion that *Zp* genes are active only during oogenesis, therefore, it is justified to keep an open mind towards alternative possibilities. We too have observed anomalies during the past 10 years, for example: repeated measurements of abundant ZP proteins found inside blastocysts [11, 12]. We, therefore, designed this study to evaluate the possibility that ZP2 might operate hitherto unsuspected internal roles during embryogenesis.

We show here that there is a highly abundant intracellular i.e. non-secreted fraction of ZP2 that persists during preimplantation and contributes to the timing of the morula-to-blastocyst transition as well as to the blastocyst quality in mice. After ovulation and fertilization, ZP2 did not decline monotonically during embryogenesis as expected of a maternally deposited protein, but remained among the top 1st − 5th percentile of abundance ranking at every stage examined, and even showed an apparent increase at the 8-cell / morula stage, as revealed independently by mass spectrometry (MS) and monoclonal immunofluorescence (IF). The latter also suggested that ZP2 was, in part, located outside the secretory pathway, as indicated by basic colocalization analysis with markers of Golgi apparatus and endoplasmic reticulum (ER). To determine the functional significance of intracellular ZP2 we performed protein immunodepletion using the method of ‘*Trim-Away*’ [13], which was combined with morpholino [14] to preclude resynthesis during the experiment (see Results). Given the high abundance of ZP2, the depletion stalled at ≈ 50% despite pushing *Trim-Away* to the boundaries of its technical possibilities. Yet this knockdown (KD) was associated with embryo retardation at the morula stage, suggesting a functional contribution of intracellular ZP2 to the morula-to-blastocyst transition. Resultant blastocysts presented a reduction in the size of the extraembryonic compartments (trophectoderm, primitive endoderm) as well as reduction in the rates of postimplantation development. These effects were not reproduced when the *Trim-Away* cocktail was redirected to an irrelevant protein (Green Fluorescent Protein, GFP), supporting specificity. Global gene expression analysis of ZP2 KD embryos followed by gene ontology (GO) analysis returned significant terms in the transcriptome that are fully consistent with the observed phenotypes; whereas in the proteome, the significantGO terms expose a defect in protein synthesis, which we confirmed using the orthogonal assay of O-propargyl-puromycin (OPP) incorporation into nascent proteins.

These novel findings are not the end of a story, but rather a beginning. They bring up new awareness about the functional biology of ZP proteins, expanding their original definition of extracellular-operating, oocyte-specific gene products. In fact, ZPs persist in mouse embryos until at least the blastocyst stage, and operate functionally relevant intracellular roles. In addition to the three canonical functions of ZP proteins (1, oocyte encasement in the ovarian follicle; 2, sperm-oocyte interaction; 3, physical protection of the preimplantation embryo) and the more recently proposed 4th function of ZP3 [15], we show here that there is a hitherto unrecognized intracellular requirement for ZP2 during the morula-to-blastocyst transition (a putative 5th function), in mice. We will discuss why intracellular ZP2 was overlooked in earlier work, why a protein of the secretory pathway like ZP2 was found where it should not be, and why the phenotype of the ZP2 protein KD presented here is different from that of the original *Zp2* locus deletion study. To conclude, we will prompt a reflection on the definition of oocyte-specific genes, given that the previous membership of *Zp* genes was based on gene transcription, which now needs to be revisited in the light of protein data.

## Methods

### Mouse embryo production

All mice used in this study were reared in-house and used at the MPI Münster in accordance with the standardized protocols and workflows used in nearly all publications of this group. The mice were maintained in individually ventilated type 2 L cages (EHRET GmbH Life Science Solutions, 79111 Freiburg, Germany), with autoclaved Aspen wood as bedding material and a cardboard tube as enrichment, in groups of five females or individually as males. Access to water (acidified to pH 2.5) and food (Teklad 2020SX, ENVIGO RMS GmbH, Düsseldorf, Germany) was *ad libitum*. The animal room was maintained at a controlled temperature of 22 °C, a relative humidity of 55%, and a 14/10 h light/dark photoperiod (light on at 6:00 a.m.). The hygiene status was monitored every 3 months, as recommended by Federation of European Laboratory Animal Science Associations (FELASA), and the sentinel mice were found free of pathogens that might have affected the results of this study. Ovulation was induced by intraperitoneal (i.p.) injection of pregnant mare’s serum gonadotrophin (eCG) (Pregmagon, IDT, 06861 Dessau-Roßlau, Germany) and hCG (Ovogest, MSD Tiergesundheit, Unterschleißheim, Germany). Lean B6C3F1 females aged 8–10 weeks and weighing approx. 25 g were injected i.p. with 10 I.U. eCG (5 pm), and then 10 I.U. hCG 48 h later (5 pm). The females were mated to CD1 studs aged 5–7 months to produce zygotes, or left unmated to collect MII oocytes. On the next morning, the females were euthanized by cervical dislocation, according to Annex IV of Directive 2010/63/EU. Euthanasia was performed on conscious animals by personnel trained and assessed as competent for this procedure, causing immediate loss of consciousness followed by death. No anesthetic or sedative was administered beforehand; this was a deliberate choice, because exposure of the genital tract to inhaled or injected anesthetic agents can alter the quality of oocytes and embryos under study [16–20], and a rapid, drug-free cervical dislocation avoids such confounding effects on the very specimens being analyzed. The cumulus-oocyte complexes were recovered, dissociated in hyaluronidase (cat. no. 151271, ICN Biomedicals, Costa Mesa, CA, USA; 50 I.U./mL in HCZB), dissolved in Hepes-buffered Chatot, Ziomek and Bavister medium (HCZB) with bovine serum albumin (BSA; Probumin, Millipore, Kankakee, IL, USA) replaced by polyvinylpyrrolidone (PVP, 40 kDa; cat. no. 529504, Calbiochem, EMD Biosciences, La Jolla, CA, USA) 0.1% w/v. The cumulus-free zygotes were cultured in 500 µL of Potassium (K) simplex optimization medium (KSOM) prepared in house as per original recipe [21]. KSOM contained free amino-acids (aa) both essential and non-essential (hence called KSOM(aa)), 0.2% (w/v) BSA and gentamicin (50 µg/mL), and was used in a four-well Nunc plate without oil overlay, at 37 °C under 6% CO_2_ in air.

### Proteome analysis of all preimplantation stages in data-dependent acquisition (DDA) mode

This method was conducted essentially as previously described [15]. Embryos were collected for proteome analysis at consecutive stages up to blastocyst (*n* = 100 embryos per sample). The zonae were either left in place or removed with acidic Tyrode’s solution. Embryos were lysed in PreOmics buffer (as part of the kit iST 8x; Preomics Planegg/Martinsried Germany) and lysates were processed using the Preomics iST kit according to the manufacturer’s instructions (PreOmics, 82152 Planegg-Martinsried, Germany). The digested and purified samples were subsequently dried in an Eppendorf Concentrator and resuspended in Buffer A (0.1% formic acid) for the liquid chromatography–mass spectrometry (LC-MS/MS) measurement. Nanoflow reversed-phase liquid chromatography was performed using a nanoElute 2 UHPLC system (Bruker Daltonics, Bremen, Germany), coupled online to a timsTOF Pro2 mass spectrometer via a CaptiveSpray nano-electrospray ion source (Bruker Daltonics). Peptide mixtures were chromatographically separated either on a 40 cm long fused silica emitter (CoAnn Technologies, Richland WA 99354, USA) home-packed with 1.5 μm ReproSil Saphir 100 C18 beads (Dr. Maisch, 72119 Ammerbuch-Entringen, Germany) or on a 50 cm long PepSep column (Bruker) via a multisegment gradient from 5% Buffer B (80% acetonitrile, 0.1% formic acid) to 18% B within 57 min, from 18 to 27% B in 21 min, and from 27 to 37% B in 13 min, before being flushed with 90% B (flow rate 300 nl/min). The column temperature was maintained at 50 °C. The timsTOF was operated in the DDA mode using the DDA-PASEF standard_1.1_cycletime conditions (Bruker Daltonics). Raw data were processed for identification and quantification by MaxQuant Software (version 2.0.3.0; [22]) with the ‘iBAQ’ option enabled and the ‘requantify’ option disabled. The search for identification of peptides was performed against the UniProt mouse database (version from 04/2019) concatenated with reversed sequence versions of all entries and supplemented with common laboratory contaminants. Parameters defined for the search were trypsin as the digesting enzyme, allowing two missed cleavages, a minimum length of six amino acids, carbamidomethylation at cysteine residues as a fixed modification, oxidation at methionine, and protein N-terminal acetylation as variable modifications. The maximum mass deviation allowed was 20 ppm for the MS and 0.5 Da for the MS/MS scans. Protein groups were regarded as identified with a false discovery rate (FDR) set to 1% for all peptide and protein identifications; only proteins with at least 2 identified unique peptides were considered further. The molar fractional content of each protein P in a sample (relative iBAQ intensities = riBAQ_P_) was determined as originally described in [23] and subsequently applied in [24], as follows:

$${\mathrm{riBAQ}}=\frac{{\mathrm{iBAQ}}_{\mathrm{i}}}{\sum^{n}_{{\mathrm{i}}=1}{\mathrm{iBAQ}}_{\mathrm{i}}}$$

The mass spectrometry proteomics data have been deposited in the ProteomeXchange Consortium via the PRIDE repository (http://proteomecentral.proteomexchange.org), with the dataset identifier PXD056725.

### Evacuation of zonae for immunoblot analysis

This method was conducted essentially as previously described [15]. Oocytes were transferred in groups of 10 to a micromanipulation drop on the stage of a Nikon Eclipse TE2000-U inverted microscope fitted with Nomarski optics and Narishige micromanipulator. The micromanipulation medium consisted of HCZB + 0.2% (w/v) PVP. Each oocyte was held firmly in place with the holding needle by applying negative pressure (suction). An Eppendorf’s TransferTip (ES) needle operated by an Eppendorf’s CellTram Vario (Eppendorf, Hamburg, Germany) was used to pierce through the zona and aspirate the oocyte. The evacuated zonae were concentrated on the bottom of a tube by centrifugation, the supernatant was replaced with RIPA buffer, and the lysate was processed further as described below.

### Synthesis and purification of ZP2 antibody

The antibody was produced in-house from hybridoma cells (ATCC’s IE-3 cell line, cat. no. CRL-2463). The anti-ZP2 IgG2a antibody was washed three times and concentrated at 4 °C using Amicon Ultra-0.5 100 kDa centrifugal filter devices (cat. no. UFC100, Merck Millipore, Darmstadt, Germany), which remove salts and impurities, allowing the antibody to be eluted in pure water.

### Immunoblotting analysis of ZP2 expression in oocytes and preimplantation embryos

Oocytes or embryos were gently centrifuged in protein-free HCZB medium at 100 g for 20 min to form a tiny pellet. The supernatant was carefully aspirated using a mouth-operated micropipette, and replaced by RIPA buffer containing protease inhibitors. The resultant lysates were mixed with 6x Laemmli sample buffer and boiled for 5 min at 99 °C. These samples were loaded and run on a 12% separation gel, next to a lane of Precision Plus Protein Kaleidoscope ladder (cat. no. 1610375, Bio-Rad, Feldkirchen, Germany), and blotted onto a PVDF membrane. The membrane was blocked for at least 3 h and incubated (3% nonfat dry milk in 0.1% PBS-Tween20) with primary antibodies overnight at 4 °C. The antibodies against ZP2 (cat. no. A10126, Abclonal; IE-3, ATCC cat. no. IE-3 – CRL-2463; 21832-1-AP, ProteinTech) were applied at a dilution factor of 1: 2000. After 3X washing for 10 min each in 0.1%PBS-Tween20, the membrane was incubated with horseradish peroxidase (HRP)-coupled secondary antibody at room temperature for 1 h. The membrane was washed and then developed with chemiluminescent HRP substrate solution. The membrane was subsequently reprobed for α-TUBULIN as a loading control. The antibody against α-TUBULIN (cat. no. T6199, Sigma-Aldrich Chemie GmbH) was applied at a dilution factor of 1: 5000. ZP2 and α-TUBULIN signals were acquired from the same membrane. The chemiluminescent signal of oocytes was detected on film using the AGFA Curix 60 to test the quality of IE-3 antibody (Supplementary Figure S1a, b); the ZP2 chemiluminescent signal of a developmental series was captured digitally at 16-bit depth using a Bio-Rad ChemiDoc™ Imager (Supplementary Figure S2a, b).

### Immunofluorescence (IF) analysis of ZP2 in oocytes and preimplantation embryos

This method was conducted essentially as previously described [15]. Oocytes or embryos were analyzed by performing an immunostaining followed by confocal microscopy imaging, as per routine protocol. Briefly, embryos were fixed with 1.5% formaldehyde in 1x PBS for 20 min at 4 °C, permeabilized with 0.1% Triton X-100 in 1x PBS for 20 min at 4 °C, and then blocked with 2% BSA, 2% glycine, 5% donkey serum, 0.1% Tween20 in 1x PBS overnight at 4 °C. When applicable, the zona was removed by bathing the oocytes or embryos in acidic Tyrode’s solution (cat. no. T1788, Sigma-Aldrich Chemie GmbH, 82024 Taufkirchen Germany) prewarmed at 30 °C. The primary anti-ZP2 antibody was monoclonal IgG raised in rat-mouse hybridoma (CRL-2463, ATCC IE-3 [25, 26]; Supplementary Figure S1a, b) applied at 1:200 overnight at 4 °C. An appropriate Alexa Fluor-tagged secondary antibody (Invitrogen, Thermo Fisher Scientific, Karlsruhe, Germany) was matched to the primary and incubated for 1–2 h at room temperature. Direct labeling of ZP2 antibody: Hybridoma anti-ZP2 antibody ATCC IE-3 CRL-2463 was used for direct labeling. The labeling was performed after the instructions of the Alexa Fluor^®^ 647 Conjugation Kit (Fast)- Lightning-Link^®^ (Abcam cat. no. ab269823). DNA counterstaining was performed with YO-PRO-1 or Hoechst 33342 (1 µM). For imaging, embryos were placed in 5 µl drops of PBS on a 50-mm thin-bottom plastic dish (Lumox hydrophobic dish, Greiner Bio-One, Frickenhausen, Germany) and overlaid with mineral oil (cat. no. M8410, Sigma-Aldrich Chemie GmbH, 82024 Taufkirchen, Germany). Images were captured using a 20x CFI Plan Apochromat VC objective on an inverted motorized Nikon TiE2000 microscope fitted with an Andor Dragonfly 502 spinning disc confocal unit Scanning System. Optical sections per embryo were captured using a high resolution (2048 × 2048 pixel) sCMOS ANDOR camera. Maximum projections were analyzed with Fiji [27].

### Subcellular and colocalization analysis of ZP2

The analysis of ZP2 subcellular distribution in preimplantation mouse embryos was performed by direct and indirect IF. Embryos were fixed with 1.5% formaldehyde in 1x PBS for at least 30 min at 4 °C. Then they were permeabilized with 0.1% Triton X-100 in 1x PBS for 20 min at 4 °C and blocked with 2% BSA, 2% glycine, 5% donkey serum and 0.1% Tween20 in 1x PBS overnight at 4 °C. After blocking, the zona was removed by extensive washing in warm acidic Tyrode’s solution, which may take several minutes due to the prior fixation. For the direct and indirect IF, the primary ZP2 antibody ATCC IE3 CRL-2463 was applied at 1:200 and incubated overnight at 4 °C. Hoechst 33342 was used for DNA counterstaining. Super-resolution images were captured by using a 20x Plan-Apochromat objective on a laser scanning confocal microscope (LSM980) with the Zeiss Zen (Zeiss Zen version 3.5) software. Images were acquired using Airyscan 2 detector. The analysis of ZP2 colocalization was conducted against markers of two compartments of the secretory pathway, as previously described [15]. Antibody directed against Golgi matrix protein GM130 (cat. no. 610823, BD Bioscience, Heidelberg, Germany) and antibody directed against the ER luminal chaperone Calreticulin (cat. no. PA3-900, Thermo Fisher Scientific, Karlsruhe, Germany) were applied and detected following the same protocol as described in “*Immunofluorescence (IF) analysis of ZP2 in oocytes and preimplantation embryos*”. The ‘coloc2’ plug-in in Fiji was used to calculate the colocalization ZP2-GM130 and ZP2-Calreticulin. Specifically, Pearson correlation coefficient and van Steensel cross-correlation function were used. The former measures the overlap of the intensities of the two signals. In the latter, one channel is displaced laterally in one-pixel steps and the correlation is recomputed at each displacement. Analysis was restricted to the cytoplasmic compartment, excluding the cortical rim in which both channels carry signal irrespective of overlap.

### Synthesis and purification of *mCherry-Trim21 *mRNA

This method was conducted essentially as previously described [15]. mRNA was synthesized from an expression construct (pGEMHE-*mCherry*-*trim21* plasmid, Addgene Plasmid #105522). The plasmid was linearized with SwaI (cat. no. FD1244, Thermo Fisher Scientific, Karlsruhe, Germany). Capped mRNA was synthesized with T7 polymerase (Ambion mMessage mMachine T7 kit) according to manufacturer’s instructions. The *mCherry-Trim21* mRNA was purified with Quick-RNA MicroPrep (cat. no. R1051, Zymo Research, 79110 Freiburg, Germany) and stored in MilliQ water at -80 °C.

### Synthesis of morpholino oligonucleotides

Translation-blocking morpholino oligonucleotides, 25 nucleotides in length, were purchased from Gene Tools, LLC (Philomath, OR 97370, USA). They were dissolved in MilliQ water at 1 mM and aliquoted before storing frozen. Sequences were selected by design parameters according to the manufacturer’s recommendations, namely approximately 50% G/C and A/T content with no predicted hairpins. Morpholinos were designed for blocking translational initiation by binding to sequences flanking the AUG initiating methionine of the two isoforms of the *Zp2* gene (Ensemble accession number ENSMUST00000033207.6 (*Zp2-201*) and ENSMUST00000208874.2 (*Zp2-205*)). The following oligonucleotide sequences were used. Morpholino targeting *Zp*2: 5‘–CTCTGCCACCTCGC*CAT*GTTGGAAG–3‘, suited to bind both *Zp2* isoforms and almost identical to the oligo previously used to inhibit ZP2 protein synthesis during oogenesis (5‘–CCACCTCGC*CAT*GTTGGAAGGTAC–3‘; [28]); morpholino targeting *Gfp*: 5‘–ACAGCTCCTCGCCCTTGCTCAC*CAT*–3’. The CAT sequence complementary to the start codon is underlined in the case of a molecule binding to a sequence containing the AUG. Using homology searches (BLAST search), each sequence was also systematically tested for possible interaction with other sequences within the mouse genome, finding none except the *Zp2* gene.

### Knockdown (KD) of ZP2 using *Trim-Away*combined with morpholino

This method was conducted essentially as previously described [15]. Zygotes were microinjected with a cocktail of *mCherry-Trim21* mRNA, Oregon Green dextran beads (OGDB), anti-ZP2 hybridoma antibody and *Zp2* morpholino at approximately 16 h post-hCG. Concentrations in the mixture were 0.15 mg/mL mRNA, 0.017 mg/mL OGDB, 4 mg/mL antibody, and 0.01 mM morpholino, respectively, in MilliQ water. This *Trim-Away* cocktail is expected to target ZP2 and result in its KD. As mock control, zygotes were microinjected with a cocktail of *mCherry-Trim21* mRNA, OGDB, anti-GFP antibody (cat.no. G10362, Thermo Fisher) and anti-*Gfp* morpholino (same concentrations as for ZP2 KD). This control *Trim-Away* cocktail cannot target ZP2, but at the same time it subjects the zygotes to all steps of the experimental procedure except for the specific active variable; hence the qualification as ‚mock KD‘. In addition, as a further control, zygotes were microinjected with a mixture of *mCherry-Trim21* mRNA and OGDB, forming a micromanipulation control (MC) to account for the possibility that mRNA overexpression itself could perturb cellular proteostasis. Microinjections were conducted on the stage of a Nikon TE2000U microscope fitted with a PMM-150 piezo drill (PrimeTech, 635 Nakamukaihara Tsuchiura Ibaraki 300–0841 Japan), using a blunt-end glass needle designed for intracytoplasmic sperm injection – ICSI (inner diameter 6–7 μm, outer diameter 8–9 μm) filled with 2–3 µl mercury at the tip. Volumes were pressure-injected into the zygote using a Gilmont GS-1200 micrometer syringe operated manually. During the microinjection, cells were kept in a 200–300 µl drop of HCZB medium on a glass-bottomed (Nomarski optics) dish at a room temperature of 28 °C. After microinjection, zygotes or embryos were allowed to recover in the drop for 5–10 min, before returning them to KSOM(aa) medium.

### Analysis of cell lineage allocation of blastocysts

Blastocysts were fixed as described (*“Immunofluorescence (IF) analysis of ZP2 in oocytes and preimplantation embryos”)* and subjected to triple immunostaining followed by confocal microscopy imaging to identify and map the different cell lineages, as described previously [29]. The following primary antibodies were applied simultaneously to the specimens overnight at 4 °C for analysis of cell lineage allocation: anti-CDX2 mouse IgG1 (Emergo Europe, The Hague, The Netherlands, cat. no. CDX2-88), anti-NANOG rabbit IgG (Cosmo Bio, Tokyo, Japan, cat. no. REC-RCAB0002P-F) and anti-SOX17 goat IgG (cat. no. AF1924, R&D Systems) in dilutions of 1:200, 1:2000 and 1:100, respectively. Appropriate Alexa Fluor-tagged secondary antibodies (Invitrogen) were matched to the primaries and incubated for 1–2 h at room temperature. Blastocysts were imaged as described (“*Immunofluorescence (IF) analysis of ZP2 in oocytes and preimplantation embryos*”). Cells were counted in maximum projection images using Image-J.

### Outgrowth assay

Blastocysts were transferred individually onto a feeder layer of γ-ray-inactivated (30 Gy) mouse embryonic fibroblasts (C3H background) grown to confluence in 96-well plates (flat bottom), as previously described [30], with adaptations [31]. The medium consisted of high-glucose Dulbecco’s modified eagle medium with high glucose (cat. no. D5671, Sigma-Aldrich Chemie GmbH) supplemented with 15% heat-inactivated fetal bovine serum (BioWest, Nuaillé, France), GlutaMAX 1X (cat. no. 35050-038, Gibco at Thermo Scientific), penicillin/streptomycin 1X (cat. no. P4333, Sigma-Aldrich Chemie GmbH), nonessential amino acids 1X (cat. no. M7145, Sigma-Aldrich Chemie GmbH), mercaptoethanol 0.1 mM (cat. no. 31350-010, Gibco at Thermo Scientific) and 1000 Units/mL LIF (produced in-house).

### Transfer of mouse embryos to the pseudopregnant females

Embryos were surgically transferred in groups of ten to the genital tract of pseudo-pregnant CD1 recipients previously mated with vasectomized CD1 males. These males were already available as standing batteries in Michele Boiani’s animal colony and were not created specifically for this project. Blastocyst-stage embryos were transferred into both uterine horns (one horn for the treated group, the other horn for control) of females that had received the copulation plug two days before. Prior to surgery, the females were anesthetized with Ketamine (80 mg/kg body weight)/Xylazin (16 mg/kg)/Tramadol (15 mg/kg) diluted in PBS, delivered i.p. A laparotomy was performed to expose the uterine horns. A glass capillary carrying the blastocysts was then inserted into the uterine cavity via a puncture made with a 27-Gauge needle. The uterine horns were then gently returned to the abdominal cavity. The surgical wound in the skin was closed with Michel suture clips. Post-surgical pain was alleviated by providing the animals with Tramadol in drinking water (1 mg/mL) for three days. Pregnancies were evaluated by Caesarean section at embryonic day 16.5 (~ 3 days before term). Recovery of the fetuses by Caesarean section at embryonic day 16.5 was a terminal procedure: the pseudopregnant recipient was deeply anaesthetized by intraperitoneal injection of the same Ketamine (80 mg/kg)/Xylazin (16 mg/kg)/Tramadol (15 mg/kg) mixture and, while unconscious, was euthanized by cervical dislocation immediately before the uterus was opened, so that the mother animal did not regain consciousness. The fetuses were sedated through the effect of the Ketamine/Xylazin/Tramadol administered to the mother, and were decapitated immediately after dissection from the uterus and counting.

### Proteome analysis of late morula-stage embryos in data-independent acquisition (DIA) mode after ZP2 KD

Morulae were collected from each group on day 3, deprived of the zona pellucida by washing in acidic Tyrode’s solution, lysed in PreOmics iST LYSE buffer and stored at -20 °C until further processing. Lysates were processed using the PreOmics iST kit according to the manufacturer’s protocol. Purified peptides were dried down in an Eppendorf Concentrator, eventually dissolved in 10 µl LC-LOAD loading buffer, of which 1 µl was injected into the mass spectrometer. Nanoflow reversed-phase liquid chromatography was performed using a nanoElute 2 UHPLC system (Bruker Daltonics), coupled online to a timsTOF Pro2 mass spectrometer via a CaptiveSpray nano-electrospray ion source (Bruker Daltonics). Peptide separation was achieved using a 25 cm × 75 μm fused silica capillary column (CoAnn Technologies), home-packed with 1.5 μm ReproSil Saphir 100 C18 beads (Dr. Maisch). To elute bound peptides a linear gradient of 4–27% buffer B (99.9% acetonitrile, 0.1% formic acid) over 60 min was applied, with buffer A consisting of 0.1% formic acid. The flow rate was set to 300 nl/min. The mass spectrometer was operated in DIA-PASEF mode using the standard DIA-PASEF long-gradient method. Signals were recorded across a mass range of 100–1700 m/z and an ion mobility range from 1/K₀ = 0.60 to 1.60 Vs/cm², with a ramp time of 100 ms in the dual TIMS analyzer. Collision energy was adjusted as a function of ion mobility, ranging from 59 eV at 1/K₀ = 1.60 Vs/cm² to 20 eV at 1/K₀ = 0.6 Vs/cm².

Raw mass spectrometry data were processed using DIA-NN software (v2.0.2) in library-free search mode with heuristic protein inference performed at the gene level. Neural networks were employed in single-pass mode, enabling matching between runs, and QuantUMS was applied as the quantification strategy. The predicted library included entries sourced from mouse UniProt UP000000589_10090_additional.fasta database (April 2024 version), including isoforms, the mCherry sequence, and common laboratory contaminants. Search parameters included carbamidomethylation of cysteine residues as a fixed modification, while oxidation of methionine and acetylation of protein N-termini were set as variable modifications. Trypsin was specified as the digesting enzyme, allowing up to two missed cleavages and peptides with lengths between 7 and 30 amino acids. Mass tolerances were set to 20 ppm for MS scans and 15 ppm for MS/MS scans, with charges above + 4 excluded from analysis. Protein groups were considered identified with a false discovery rate (FDR) of ≤ 1% at both peptide and protein levels. Initial downstream analysis was performed using Perseus software (v1.6.15.0). Common laboratory contaminants were removed from the dataset, and protein intensity values provided in the pg_matrix.tsv table were log₂-transformed. The mass spectrometry proteomics data have been deposited in the ProteomeXchange Consortium via the PRIDE repository (http://proteomecentral.proteomexchange.org), with the dataset identifier PXD062983.

### Transcriptome analysis of blastocyst-stage embryos after ZP2 KD

Sequencing and raw data processing was provided by the Core Genomic Facility of the Faculty of Medicine of the University of Münster, as described in this paragraph. RNA was extracted and purified using Quick-RNA MicroPrep (cat. no. R1051, Zymo Research Europe GmbH). The library preparation of the total RNA was performed with the Watchmaker mRNA Library Prep Kit (7K0078-024) according to the manufacturer’s instructions. Paired-end sequencing with a read length of 111 bp was performed on NovaSeq^®^ X Plus System using the corresponding NovaSeq^®^ X Series 1.5B Reagent Kit (200 Cyc). Total RNA integrity and quality of the library were assessed using a TapeStation4200 (Agilent, Santa Clara, CA, USA). On average, the libraries contained 24.7 ± 2.9 million 111-base paired-end reads. Using a molecular barcode, the samples were demultiplexed (DRAGEN BCL Convert 4.3.13) to fastq data and quality controlled (FastQC version 0.12.1 [32]). Trimmomatic ([33]; version 0.39) was used for adapter trimming and read filtering. Reads were aligned to the reference genome (*Mus musculus* Ensembl GRCm39) using Hisat2 ([34]; version: 2.2.1). Aligned reads were sorted using samtools ([35]; version 1.16.1). Sorted and aligned reads were counted into genes using HTSeq framework ([36]; version 2.0.3), then these counts were used to calculate the FPKM (Fragments Per Kilobase of exon per Million mapped fragments) to normalize for sequencing depth and gene length. StringTie version 3.0.0. was used to calculate the potential transcripts. FPKM values for Ensembl gene identifiers corresponding to the same gene symbol were averaged, and the values associated with each gene symbol were averaged across replicates. The RNA-seq data have been deposited in the ArrayExpress repository, with the dataset identifier E-MTAB-16339.

### Reanalysis of public ribosome-profiling, translatome and proteome datasets

Processed ribosome-profiling data for mouse oocytes and preimplantation embryos were obtained from [7] and [8], and the ultra-low-input human translatome from [37]. For each dataset, ribosome-protected-fragment signal was taken as provided by the original authors without renormalization, and each gene was assigned a percentile rank against all genes detected at the same stage within the same dataset, so that stages are ranked independently and no value is normalized to the oocyte. Translation efficiency, where reported by the original study, was taken as the ratio of ribosome-protected to total RNA signal and expressed as a fold difference from the genome median of the same stage. Because each dataset had its own gene universe, sequencing depth and normalization, percentiles were within-dataset ranks and were not compared across datasets; the reference set for each panel is stated in the corresponding legend, together with whether the zona pellucida had been removed before analysis (Supplementary Figure S3). Published preimplantation proteomes used for comparison were [9, 12] and the human dataset of Rai et al. [38]. Candidate ZP2 interactors were retrieved from BioGRID [39] in March 2026 with no filtering other than the removal of self-interactions. Percentile computations were performed in JMP v.18.

### Principal component analysis (PCA) and over-representation analysis of differentially expressed mRNAs and proteins

RNA sequencing (RNA-seq) data analysis was performed in-house using the output of the NovaSeq^®^ X Plus System, exported to Microsoft Excel (Microsoft Deutschland GmbH, 80807 München, Germany) format and imported in JMP Pro v. 18 (JMP Austria, Germany & Switzerland, 69188 Heidelberg, Germany). Proteome data analysis was performed in-house using the output of the MaxQuant and DIA-NN exported to Microsoft Excel. For the PCA, the transcripts or proteins were considered that were present in all samples of all replicates. DIA intensities and FPKM values were Z-normalized, whereby features were mean-centred and scaled to unit variance across samples, and the PCA was computed on the resulting matrix in JMP v.18. Calls of differentially abundant proteins (DAPs) and differentially expressed genes i.e. mRNAs (DEGs) were made by a two-step approach, using raw p-values and fold changes first, and then by intersecting the items using Venn diagrams, so as to narrow down the list to those DAPs and DEGs found exclusively in ZP2 KD or in mock KD. The nominal thresholds were used only as a candidate-selection filter for the Volcano plots and the Venn intersections; they were not be used to make claims about individual proteins or transcripts. Formal multiple-testing control was applied at the next step, the over-representation analysis, where terms were reported only at Benjamini–Hochberg FDR ≤ 0.05. Over-representation analysis was performed with the WEB-based GEne SeT AnaLysis Toolkit (*Webgestalt*) [40, 41]. Terms with a FDR ≤ 0.05 in the ontology ‘Biological process’ (BP) were considered enriched. Because the input lists are selected without correction from three to four replicates per group, individual DAP and DEG calls should be regarded as exploratory, and the biological conclusions of this section rest on the FDR-controlled enrichment rather than on any single gene or protein.

### OPP assay for detection of *de novo*protein synthesis

This method was conducted essentially as previously described [15]. Embryos were sampled at each developmental stage (zygote, 2-cell, 4-cell, 8-cell, morula, blastocyst) and incubated with 20 µM O-propargyl-puromycin (OPP) dissolved in KSOM(aa) medium for 3 h. As negative controls, embryos were either precultured in 50 µg/ml cycloheximide for 2 h before applying OPP, or were not processed for detection (see below). The first control validates that the OPP signal is due to protein synthesis; the second control is needed for background subtraction during quantification. Treated and control embryos were fixed with 3.7% formaldehyde for 30 min and permeabilized with 0.5% Triton X-100 for 20 min. For detection of the OPP, embryos were incubated with the CLICK reaction cocktail containing the AZDye555-Azide (prepared according to the protocol from Jena Bioscience) for 60 min at RT, protected from light. All reagents were then washed off in a large excess volume of PBS, and the specimens were subsequently counterstained with Hoechst 33342. For imaging, embryos were pipetted into a 3 µl drop of PBS overlaid with oil on a glass-bottomed petri dish. Images were taken on a Nikon TE2000-U microscope fitted with a high resolution (2048 × 2048 pixel) sCMOS ANDOR camera. Fluorescence intensities were measured using Image-J, and the intensities of the second control were subtracted from those of the OPP.

### Statistical analysis of developmental rates and IF images

The assumption of non-normality was applied to most datasets. Therefore, developmental rates of embryos, image intensities of IF, and transcript and protein levels of ZP2 KD, mock KD and MC were analyzed non-parametrically by Wilcoxon rank-sum (Mann–Whitney) test, briefly ‘Wilcoxon’, using the statistical program JMP v.18 (JMP Austria, Germany & Switzerland, 69188 Heidelberg, Germany). The t-test was used only for the Volcano plots of the proteome and transcriptome analyses, the Fisher exact test was used only for the histograms of the post-blastocyst developmental rates.

### Use of large language models

Claude (Anthropic) was used as a computational assistant for the re-analysis of published datasets (Supplementary Figure S3) and for the analysis of ribosomal and stress control gene sets among the differentially abundant proteins or transcripts (Supplementary Table S14). No data were generated by the model. All output was verified by the authors, who take full responsibility for the content of this article.

## Results

### Intracellular ZP2 is revealed by zona stripping and persists during preimplantation development

For 10 years, large amounts of ZP proteins have been detected in mass spectrometric (MS) analyses performed in this laboratory, despite the fact that the embryos had been deprived of the zona with acidic Tyrode’s solution prior to MS analysis [11, 12]. The anomaly had gone unnoticed in embryo studies of other investigators, because those embryos were processed with the intact zona (which obscured the additional internal component) or because the studies were not specifically interested in ZP proteins, so they did not attach importance to the fact that ZP proteins continued to be detected even though the zona had been removed [8]. Therefore, the present study was undertaken to clarify if the intracellular amounts of ZP proteins are passive leftovers from oogenesis, in which case ZP proteins should decrease monotonically from oocyte to blastocyst, as would be expected of a maternal protein legacy [4, 42]. MS and the orthogonal method of IF with monoclonal antibody were applied to examine naturally fertilized mouse oocytes (zygotes) for expression of ZP proteins and particularly ZP2. Zygotes were produced on a B6C3F1 × CD1 background, chosen for its robust developmental competence and for the uniformity of the oocytes (B6C3F1 being an F1 hybrid of inbred strains). Because the paternal contribution is outbred, this scheme carries more genetic heterogeneity than a fully inbred cross, and this is expected to increase biological variance within groups. It cannot, however, account for differences between treatments.

**Fig. 1:**
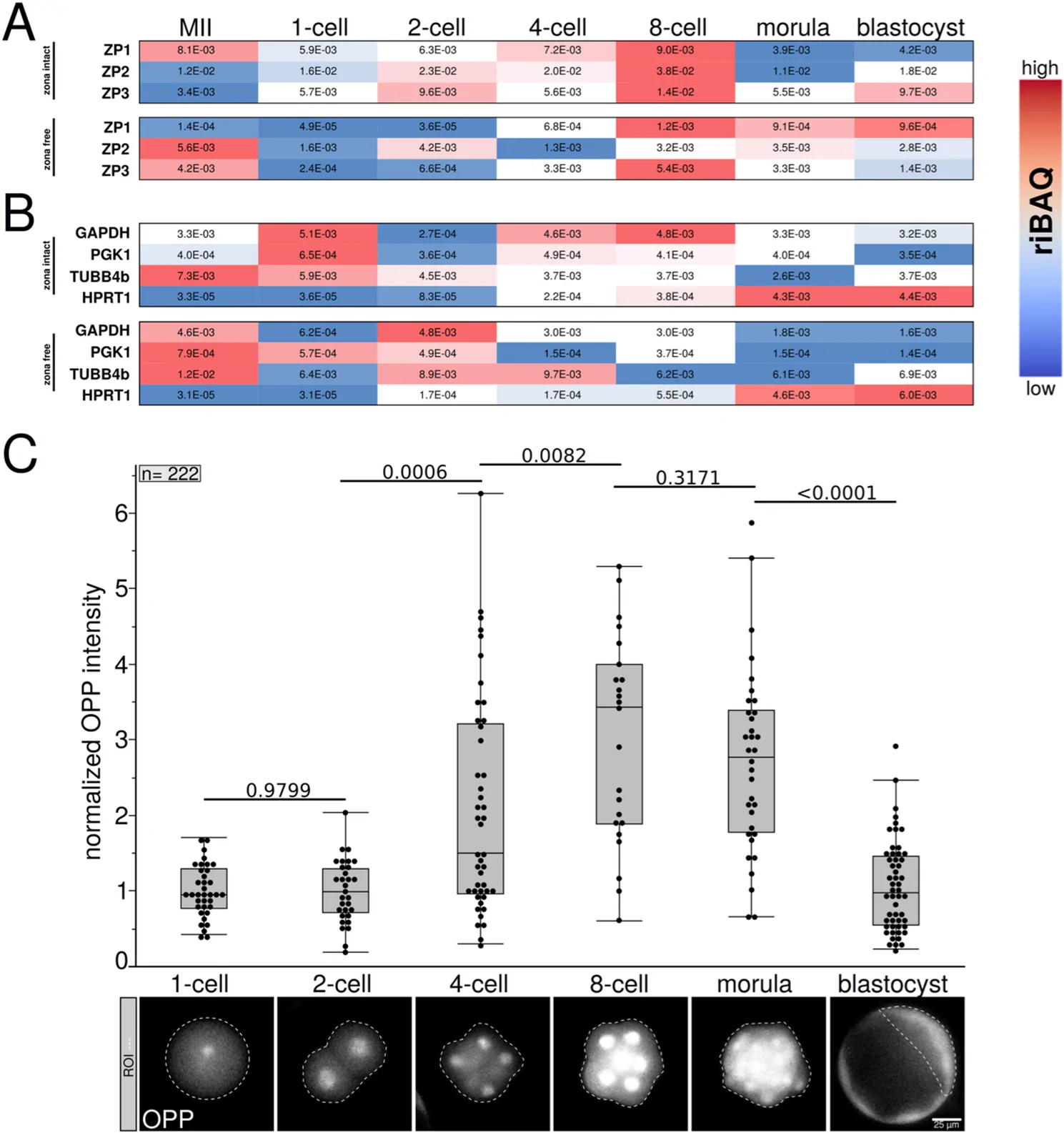
ZP protein levels and global protein synthesis during mouse preimplantation development. Mouse embryos were collected at consecutive stages of preimplantation development in vitro after culture in KSOM(aa) medium, and subjected to LC-MS/MS. Zona pellucida was either left in place (zona-intact) or removed with acidic Tyrode’s solution (zona-free). Shown in the heatmap are the relative iBAQ (riBAQ) values of protein abundance for ZP proteins (**A**) and housekeeping proteins (**B**). The color coding, indicative of expression level, was applied in each row independently of the other rows. Numbers written in the colored cells are the actual riBAQ values. The raw proteomic data are available in PRIDE repository with accession number PXD056725 and are provided in simplified form in Supplementary Table S1. **C** To understand whether the peak levels of ZP proteins at the 8-cell stage are specific or reflect an increase in overall protein synthesis, the latter was quantified using the OPP assay i.e. immunofluorescence detection of OPP incorporation in nascent protein synthesis. Representative pictures are shown underneath the diagram. The OPP signal was measured only where cellular material was present (the blastocyst cavity was not included), as indicated in the R.O.I. The measurements were normalized to the zygote (set to 1). Each dot in the diagram represents a single embryo. P-values were calculated with Wilcoxon test. Abbreviations: riBAQ, relative intensity Based Absolute Quantification [23, 24]; LC-MS/MS, Liquid Chromatography with tandem mass spectrometry; MII, metaphase II oocyte; OPP, O-propargyl-puromycin; riBAQ, relative IBAQ; R.O.I.: region of interest (dashed line)

The proteomes of two developmental series (oocyte, zygote, 2-cell, 4-cell, 8-cell, morula, blastocyst) were interrogated by MS. In both series, the embryos had developed within the intact zona until the sampling. The zona was removed after collection and prior to processing for MS in one series, while it was left in situ in the other series (control) to account for the possibility that quantitative changes in intracellular ZP protein might be obscured by the high abundance of the external zona (17% of the total protein content of the mouse oocyte [43]). Zona removal was performed with warm acidic Tyrode’s solution throughout, since the alternative option of an enzymatic removal (pronase) could impinge on the very thing that is measured by MS. Proteins were quantified in data-dependent acquisition (DDA) mode using the ‘intensity-based absolute quantification’ (iBAQ) algorithm [23, 24]. iBAQ values are approximately proportional to the number of moles of protein present and thus iBAQ_i_/Σ_j_iBAQ_j_ (adimensional) is the relative molar amount of protein ‘i’ among all proteins ‘j’, called relative iBAQ, briefly riBAQ [23, 24]. riBAQ values were quantified from DDA data, because riBAQ permits comparison both of the same protein across stages and of different proteins within a stage. DDA was used for the developmental series and data-independent acquisition (DIA) was reserved for the knockdown proteome analysis. DDA using riBAQ showed that of the three ZP proteins, ZP2 is the most abundant (Fig. 1A). Statistical distribution of the embryonic proteins’ riBAQ values ranked ZP2 within the top 1–5% (i.e., 95th − 99th percentile) of all detected proteins by abundance, even in the developmental series that was measured after removing the zona (MII, 1%; 1-cell, 4%; 2-cell, 1%; 4-cell, 5%; 8-cell, 2%; morula, 2%; blastocyst, 2%). The data of both series were concordant in showing a transient, apparent increase at the 8-cell stage (Fig. 1A), which contrasts with the expected profile of a monotonically decreasing maternal gene product. This transient increase was not observed in the proteins of housekeeping genes (*Gapdh*, *Pgk1*, *Tubulin*, *Hprt*) (Fig. 1B). The full datasets are deposited (PXD056725; Supplementary Table S1). Next, an O-propargyl-puromycin (OPP) polypeptide incorporation and fluorescent labelling assay (Fig. 1C) was applied to determine if the apparent increase in ZP proteins was associated with elevated general *de novo* translation. OPP signals were detected in both the nucleus and cytoplasm, which is not surprising since some of the cytoplasmic proteins translocate into the nucleus, as reported also in other studies conducted with the OPP assay [44]. Quantification of the total OPP signal (nucleus + cytoplasm) showed an increase that peaked at the 8-cell / morula stage (Fig. 1C). The observation that ZP2 kept stable or even increased came unexpected, since an increase in total protein synthesis would, if anything, dilute the proportion of a maternal protein. This unexpected observation prompted us to seek confirmation.

**Fig. 2:**
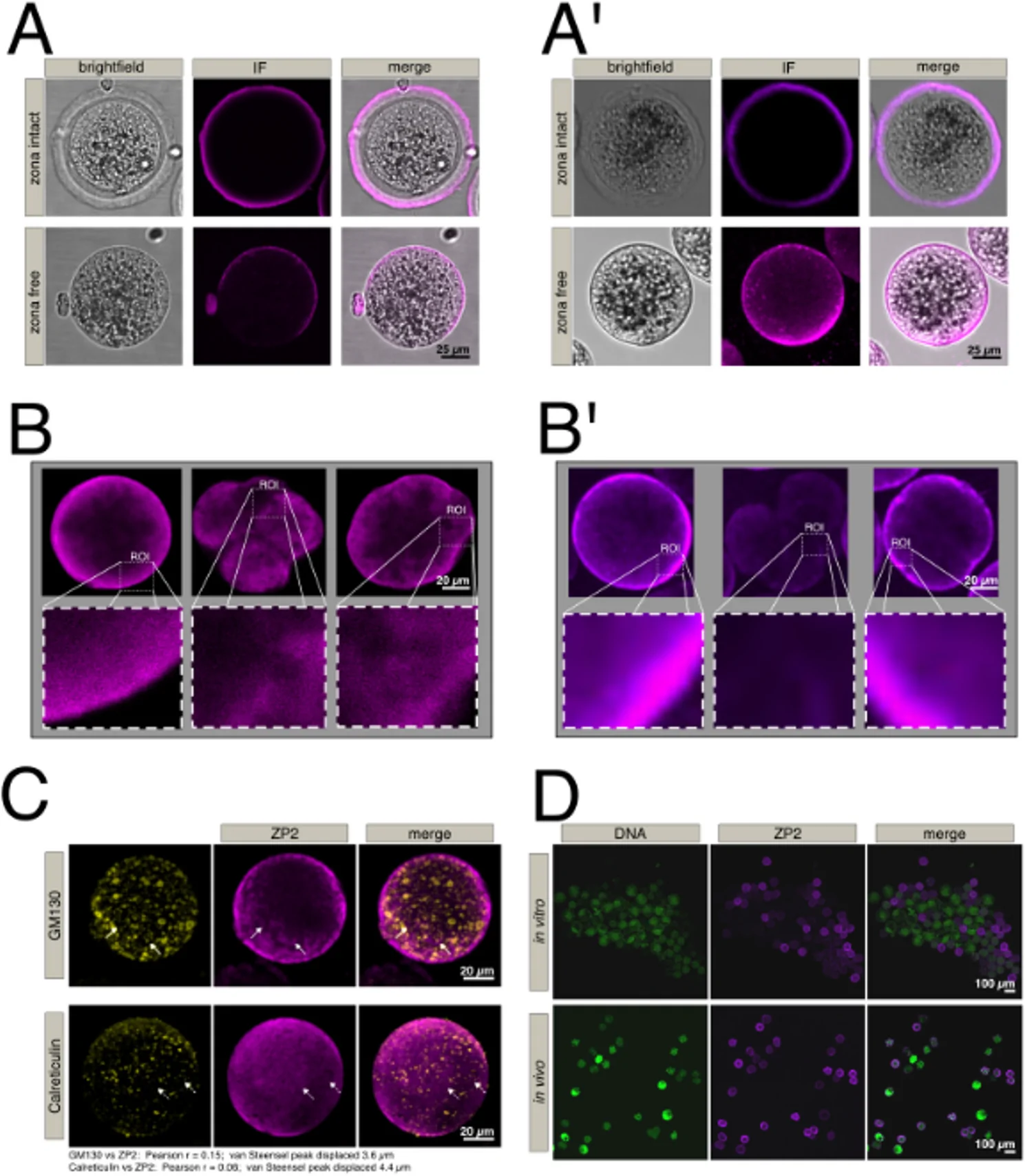
Immunofluorescence-based quantification of ZP2 in preimplantation mouse embryos cultured in vitro. **A**, **A’**. Comparison of ZP2 signal intensity and distribution in zona-intact and zona-free oocytes at the Dragonfly spinning disk microscope after indirect (**A**) and direct (**A’**) immunofluorescence microscopy using IE-3 hybridoma anti-ZP2 antibody. **B**,** B’**. Super-resolution microscopy of ZP2 at the Zeiss LSM980 fitted with Airyscan 2 detector of zygotes (left), 4-cell embryos (center) and morulae (right) after indirect IF (**B**) and direct (**B’**) immunofluorescence. The zona was removed post-fixation using acidic Tyrode’s solution. **C** Colocalization analysis of ZP2 with the cis-Golgi marker GM130 and with the ER marker Calreticulin, performed on oocytes using Fiji. Arrows indicate selected regions where the two signals are complementary rather than coincident. Pearson and van Steensel coefficients are written underneath. **D**. This panel shows all preimplantation stages within one field of view under identical acquisition settings, permitting direct comparison of signal intensities between stages, using the Dragonfly spinning disk microscope after indirect immunofluorescence with IE-3 hybridoma antibody. DNA was stained with YO-PRO-1. Abbreviations: IE-3 hybridoma antibody = anti-ZP2 (ATCC cat. no. IE-3 – CRL-2463); IF, immunofluorescence; R.O.I., region of interest

**Fig. 3:**
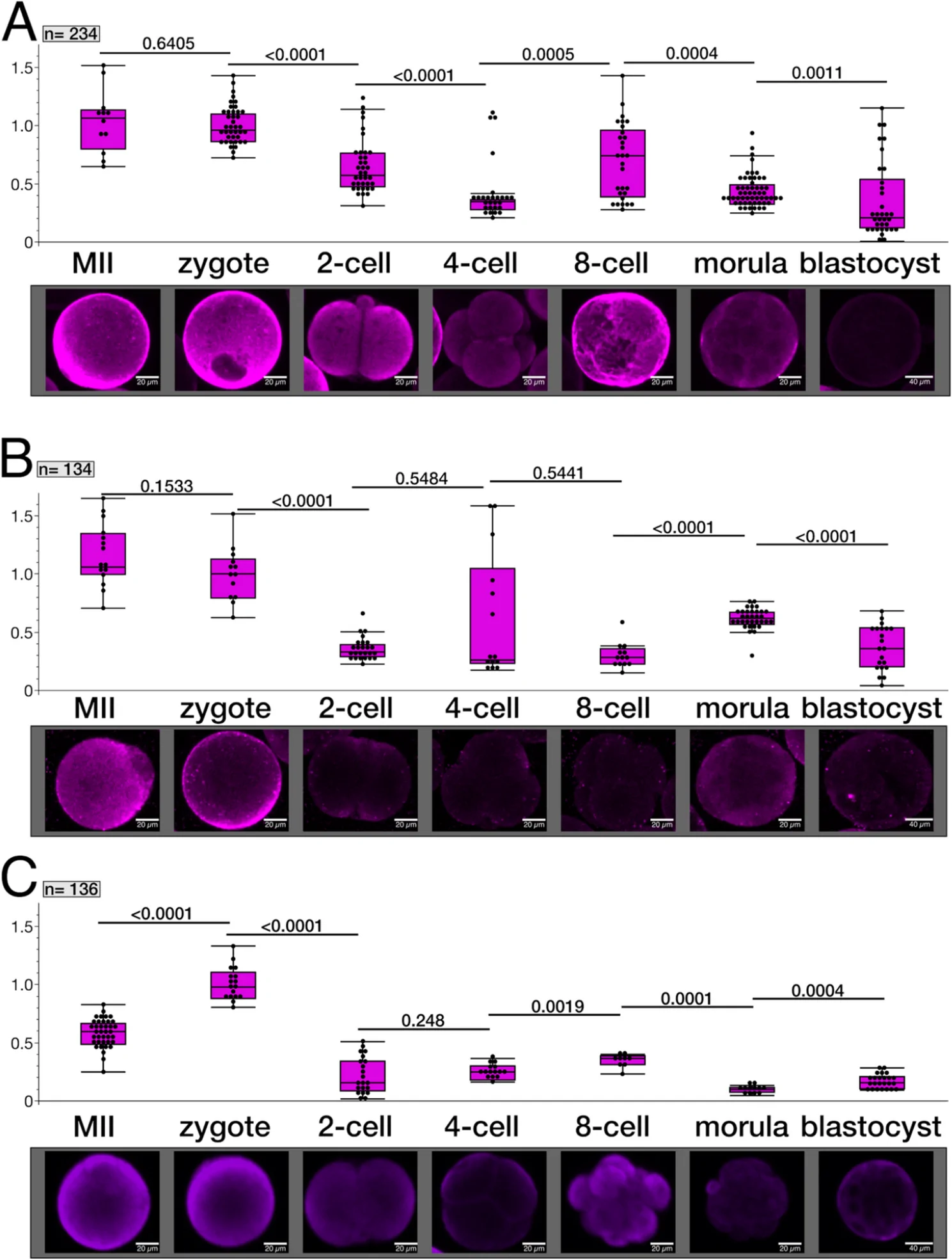
Immunofluorescence-based profiling of ZP2 protein during mouse preimplantation development. **A** Developmental series of embryos cultured in vitro and subjected to indirect IF with IE-3 hybridoma anti-ZP2 antibody. **B** Developmental series of embryos cultured in vitro and subjected to direct IF with IE-3 antibody The apparently weaker signal in (**B**) compared to (**A**) reflects direct immunofluorescence (no secondary-antibody amplification, hence lower dynamic range). **C** Developmental series of embryos obtained fresh from the genital tract without in vitro culture and examined by indirect IF with IE-3 antibody. In all three series there is an increase of ZP2 intensity, albeit not synchronous (8-cell stage in **A** and **C**, morula in **B**). The outer zona was always removed after embryo fixation and before IF. The integrated density values of the ZP2 IF intensities were quantified using Fiji, normalized to the 1-cell stage (set to 1) and plotted by stage of development. Each dot corresponds to the normalized integrated density of one embryo, and the total numbers of embryos examined across stages is shown in the top left corner of each diagram. All normalized integrated density values are provided in Supplementary Table S2. P-values were calculated with Wilcoxon test. Abbreviations: IF, immunofluorescence; MII, metaphase II (oocyte); IE-3 hybridoma antibody = anti-ZP2 (ATCC cat. no. IE-3 – CRL-2463)

The apparent increase was confirmed in ZP2 by applying the orthogonal method of IF on three additional developmental series of zygotes. In each series the zona was removed, and since zona removal method may influence antigen accessibility, fixation was conducted before applying the Tyrode’s solution and proceeding to IF. The different embryonic stages were immunostained and imaged simultaneously. A suitable antibody was identified by comparing three commercially available antibodies directed against different regions of ZP2 protein (ATCC cat. no. IE-3; ABclonal cat. no. A10126; ProteinTech cat. no. 21832-1-AP; Supplementary Figure S1a, b,). Only IE-3 met expectations in IF (e.g. stained the extracellular zona but not other cellular structures) and produced a single prominent band of the expected molecular weight in immunoblotting (Supplementary Figure S1a). This band was stronger in lysates of zona-intact oocytes compared to lysates of evacuated zonae and zona-free oocytes (with no band at the molecular weight of glycosylated ZP2 in lysates of MEFs and ES cells; uncropped full-length immunoblots are presented in Supplementary Figure S1b). In theory, IE-3 antibody could have reacted with other non-zona proteins containing the ZP domain (e.g. CUZD1, DMBT1, ENG, ERG-1, GP2, OIT3, OOSP1, PLAC1, POMZP3, TECTA, TECTB, TGFBR3, UMOD, UMODL1, UO-44, ZPLD1 [45]), but these proteins were not detected in MS datasets of this study (Supplementary Table S1). IE-3 produced the same staining after direct or indirect IF, except of course in terms of intensity (Fig. 2A), which is expected since direct IF does not amplify the signal. Here a serendipitous observation was made that would shape the course of the entire study. Leaving the zona in place (no removal with acidic Tyrode’s solution prior to fixation and IF) obscured an inner ZP2 substrate, which became overt after the zona was removed (Fig. 2A, A’). The inner ZP2 signal was further substantiated using super-resolution microscopy (Fig. 2B, B’) and basic colocalization analysis (Fig. 2C). The latter revealed only weak correlation between internal ZP2 and the cis-Golgi marker GM130 (Pearson *r* = 0.15), and even weaker with the ER marker Calreticulin (Pearson *r* = 0.06). For both markers the van Steensel cross-correlation peaked not at zero displacement but 3.6 μm (GM130) and 4.4 μm (Calreticulin) away; because displacing two genuinely overlapping signals can only reduce their correlation, a peak away from zero indicates that the signals lie adjacent to one another rather than within the same structures. Therefore, majority of the ZP2 signal seems to lie outside both of the tested compartments. Together these data define a non-Golgi, non-ER intracellular ZP2 fraction, supporting a speculation that a fraction of ZP2 lie outside the secretory pathway, as previously hypothesized on the basis of results for another ZP protein, ZP3 [15]. After conducting these checks, the developmental series was examined, capturing the different stages within a single field of view wherever possible (exemplified in Fig. 2D). One developmental series was cultured in vitro and subjected to conventional i.e. indirect IF (primary antibody + labeled secondary antibody; Fig. 3A). A second series was cultured in vitro and subjected to direct IF (with labeled primary antibody; Fig. 3B). Since culture conditions can induce gene expression artefacts [46], a third series was obtained fresh from the genital tract, without in vitro culture, and was examined by indirect IF like the first series (Fig. 3C). In all three series the signal declined after fertilization and showed an upward inflection at the 8-cell stage, followed by a decline at the blastocyst stage. An elevation extending to the morula was seen in one of the cultured series (B), whereas in the other cultured series (A) and in the freshly collected series (C) the elevation occurred at the 8-cell stage and the morula value was already declining (Supplementary Table S2). The profiles are therefore reproducible in direction but not synchronous in timing. Immunoblotting of zona-free lysates staged from MII to blastocyst (100 embryos per lane) confirmed that intracellular ZP2 protein is present during preimplantation development (Supplementary Figure S2a, b), in agreement with the MS and IF profiles.

### Protein KD shows that intracellular ZP2 contributes to the timing of the morula-to-blastocyst transition and to blastocyst quality

The function of intracellular ZP2 was determined by removing the protein itself, to see the effect, if any. It is worth recalling that embryos developed within their zona and that the removal in question concerns the intracellular ZP2 protein. To this end, the *Trim-Away* method was used, in which the protein-ubiquitin ligase TRIM21 is overexpressed from microinjected mRNA (*mCherry-Trim21*) in the presence of an antibody directed against the protein of interest. *Trim-Away* was combined with a morpholino as a precaution against re-synthesis of ZP2 protein during preimplantation. Re-analysis of two mouse ribosome-profiling datasets [7, 8] (Supplementary Figure S3) showed that *Zp2* is among the most ribosome-associated transcripts of the MII oocyte, ranking 15th of 16218 detected genes, and that it retains ribosome occupancy through the 4-cell stage; occupancy declines substantially thereafter. The morpholino was therefore expected to act during early cleavage, which is when it was administered. In the human, by contrast, all four *ZP* mRNAs remain in the top decile of ribosome-protected signal through the 8-cell stage [37]. The anti-ZP2 antibody used in *Trim-Away* was the same one that was used for IF (Figs. 2 and 3) and whose specificity was confirmed (Supplementary Figure S1a) after it had already been demonstrated for 40 years [26]. The *Zp2* morpholino target sequence overlaps with that of the antisense oligodeoxynucleotide used for thirty years to inhibit ZP2 translation during oogenesis (see Methods; [28]) and that still returns no BLAST search matches except for *Zp2*. A titration was performed to identify the minimum dose of morpholino that would counter the ZP2 increase at the 8-cell stage without preventing blastocyst formation (Supplementary Figure S4, Supplementary Table S3). Note that, given the bore of the ICSI microcapillary used for microinjection, the volume of reagents delivered (*Trim-Away*, morpholino) is estimated as ≈ 10 times greater than that commonly achieved with conventional 1 μm tip microcapillaries. As controls, TRIM21 was applied alone in the absence of antibody and morpholino, and *Trim-Away* was performed using an antibody and a morpholino that target an irrelevant protein, such as the green fluorescent protein (GFP), which is not present in the wildtype embryos of this study. These two controls address different possible sources of artefact: the TRIM21 alone receives only *mCherry-Trim21* mRNA and controls for the microinjection itself and for possible toxic effects of mRNA overexpression; whereas the mock *Trim-Away* reproduces the entire *Trim-Away* procedure but directs it against a protein that is absent from wild-type embryos (GFP), thereby controlling for antibody delivery, TRIM21 activity, and off-target effects. What these controls cannot cover is proteasomal overload, but if that were to occur, the zygotes would not progress to morula (see Discussion). In the following, these two controls are referred to as ‘micromanipulation control (MC)’ (*mCherry-Trim21* mRNA) and ‘mock KD’ (*mCherry-Trim21* mRNA + anti-GFP antibody + anti-*Gfp* morpholino). Further controls were conducted separately before the actual study began, in order to separate the contributions of the individual reagents: zygotes receiving the anti-ZP2 antibody together with *mCherry-Trim21* mRNA but no morpholino; zygotes receiving the *Zp2* morpholino alone, as well as further controls (Supplementary Figure S5). These tests showed that *Trim-Away* of ZP2 already per se affects blastocyst rates, whilst other individual treatments do not (ZP2 antibody alone; *Trim-Away* with denatured ZP2 antibody; ZP2 morpholino; *Trim-Away* of GFP).

**Fig. 4:**
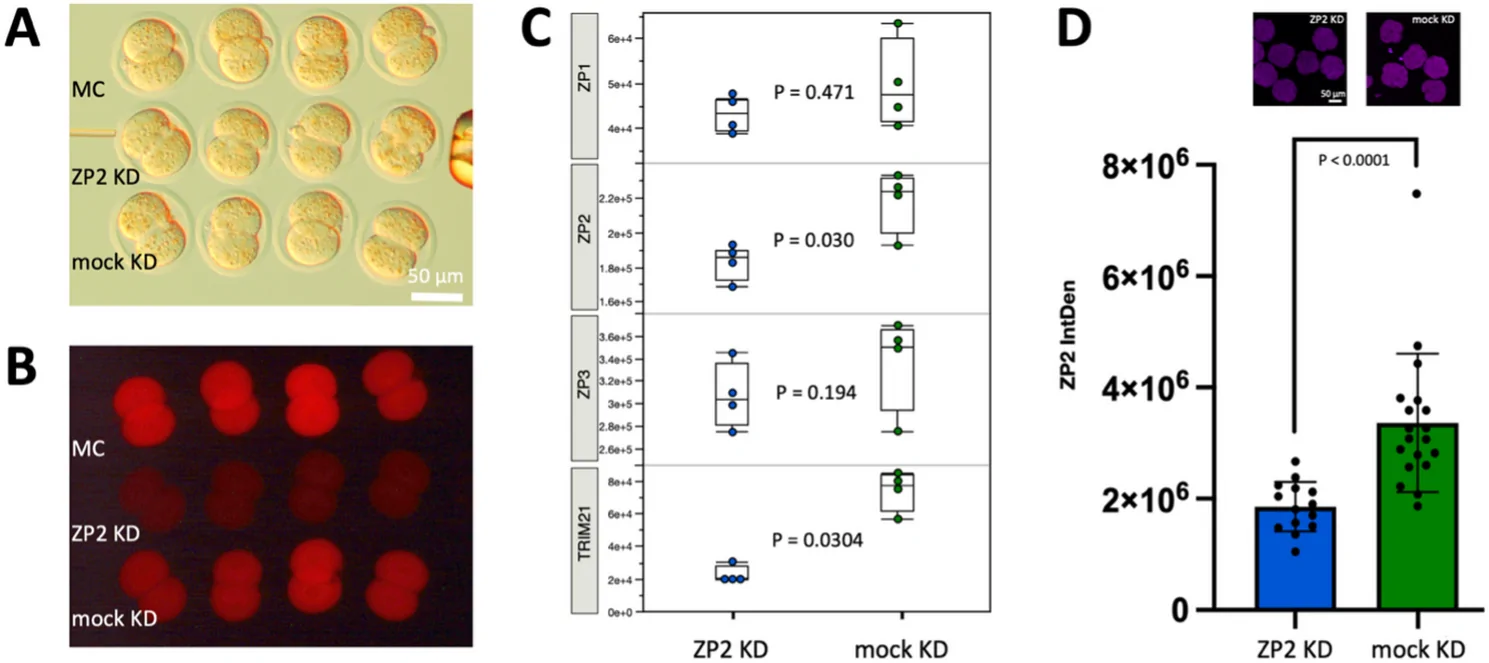
Evidence that *Trim-Away *is engaged and that ZP2 is depleted. **A**, **B** Representative images of 2-cell mouse embryos (**A**) that were microinjected the day before – as zygotes - with *mCherry-Trim21* mRNA and OGDB (micromanipulation control, MC), ZP2 KD cocktail (*mCherry-Trim21* mRNA + IE-3 hybridoma antibody anti-ZP2 + morpholino anti-*Zp2* + OGDB), mock KD (*mCherry-Trim21* mRNA + anti-GFP antibody + morpholino anti-*Gfp* + OGDB). The red CHERRY fluorescence of MC is evidence that *mCherry-Trim21* mRNA was translated (**B**). The disappearance of CHERRY fluorescence in ZP2 KD but not in mock KD is evidence that TRIM21 was selectively activated by the correct substrate (**B**). At the morula stage, protein amounts were quantified by MS in DIA-NN mode (**C**) and by direct IF (**D**). The MS values are available as dataset PRIDE PXD062983. The integrated density values of the IF intensities were quantified using Fiji and background-subtracted. Each dot in (**C**) corresponds to one measured sample (replicate), each dot in (**D**) corresponds to one embryo at the 8-cell stage, of which representative IF images are shown. Measurements presented in (**D**) values are provided in Supplementary Table S3. Both methods, MS and IF, attest to a reduction of ZP2 by ≈ 50%. P-values were calculated with Wilcoxon test. Abbreviations: IF, immunofluorescence; OGDB, Oregon Green dextran beads

To induce ZP2 KD, zygotes were microinjected with a cocktail made of *mCherry-Trim21* mRNA, ZP2 antibody and *Zp2* morpholino, as per established microinjection protocol [15, 47, 48]. The ZP2 KD experiment was flanked by the two control experiments MC and mock KD. The microinjected zygotes of the three groups were cultured in KSOM(aa) medium in parallel, and monitored daily to follow their developmental progression. TRIM21 was produced, as indicated by the accumulation of CHERRY fluorescence in MC at the 2-cell stage (Fig. 4A, B). The disappearance of CHERRY fluorescence in ZP2 KD but not in mock KD (Fig. 4A, B) indicates that TRIM21 was engaged and consumed in a substrate-dependent manner under the present experimental conditions. Independent confirmation that the consumed substrate is ZP2 was obtained by MS (Fig. 4C) and direct IF (Fig. 4D), both showing a ≈ 50% reduction in ZP2 protein at the morula stage.

**Fig. 5:**
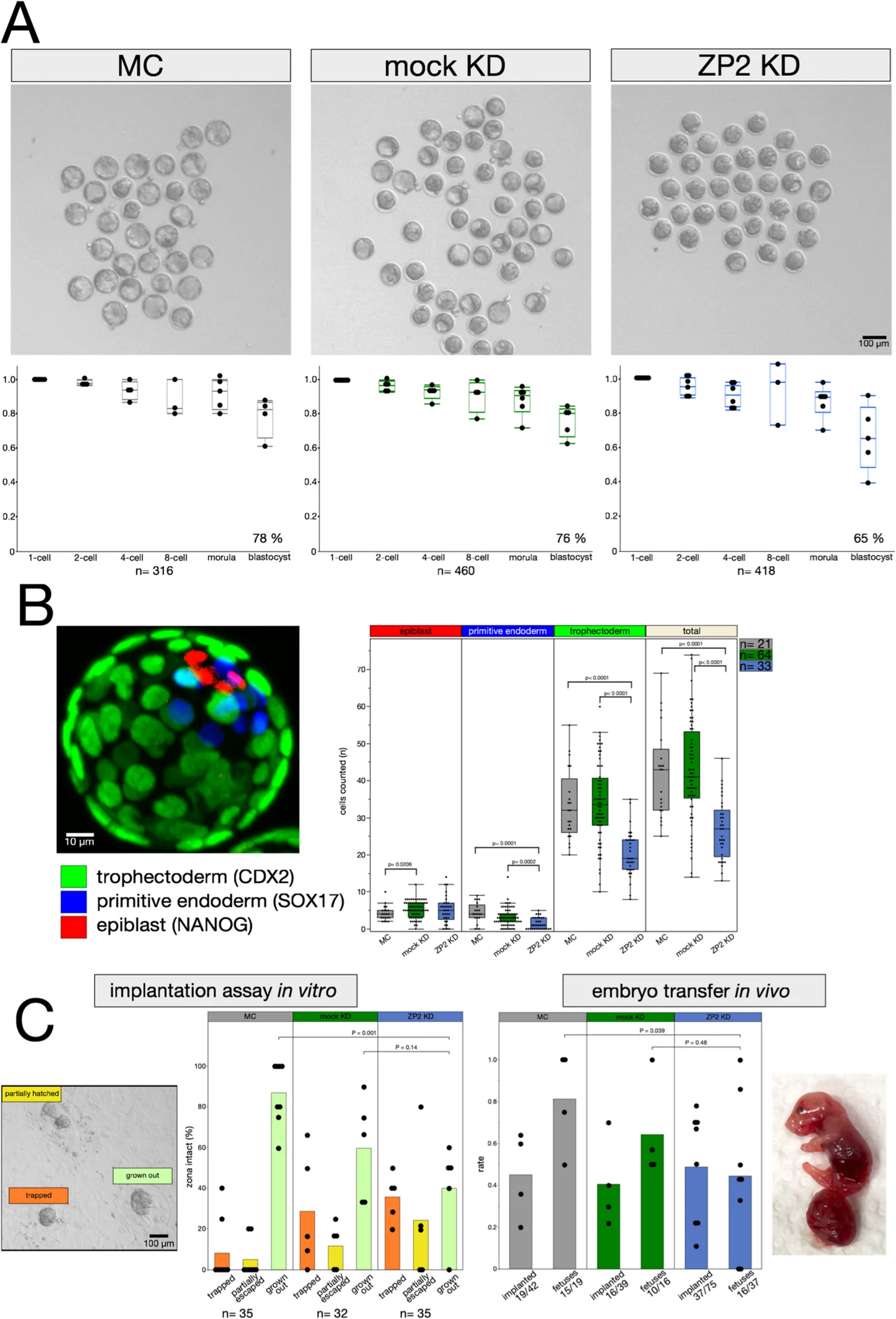
Intracellular ZP2 contributes to the timing of the morula-to-blastocyst transition and to blastocyst quality. Zygotes were microinjected with protein KD cocktail and cultured further. **A**. At the morula stage (72 h post fertilization), fewer ZP2 KD embryos had cavitated than mock KD and MC embryos at the time of scoring. The morulae eventually formed blastocysts at similar rates across the treatments. Numbers underneath the box plots are total numbers of embryos across the stages. The raw data of the developmental rates are provided in Supplementary Table S4. **B** At the blastocyst stage (96 h post fertilization), ZP2 KD embryos (*n* = 33) were deficient in trophectoderm and primitive endoderm compared to mock KD (*n* = 64) and MC (*n* = 21) embryos. The raw data of the cell counts originated from three experiments and are provided in Supplementary Table S5. **C** When examined for their ability to develop further, ZP2 KD blastocysts were less able of escaping the zona pellucida (hatching) and forming outgrowths when plated onto a fibroblast layer in vitro; in vivo, implantation rates were comparable across groups, whereas fewer implantation sites yielded a fetus after ZP2 KD than after mock KD or MC. Numbers underneath the bar plots are the blastocysts plated on feeders (implantation assay in vitro) or the fetuses obtained from the blastocysts transferred to uterus (embryo transfer in vivo). The raw data of the outgrowth and embryo transfers are provided in Supplementary Tables S6 and S7, respectively. P-values in (**A**) and (**B**) were calculated with the Wilcoxon test; proportions in (**C**) were compared by Fisher exact test on pooled counts. Numbers underneath the bar plots are the blastocysts plated on feeders (implantation assay in vitro) or, for the embryo transfer in vivo, the number of implantation sites and the number of fetuses obtained; recipients that yielded no implantation site are not represented in the in vivo denominators. Abbreviations: ZP2 KD, ZP2 knockdown by *Trim-Away* ZP2 + morpholino anti-*Zp2*; mock KD, mock knockdown by *Trim-Away* GFP + morpholino anti-*Gfp;* MC, micromanipulation control (overexpression of *mCherry-Trim21* mRNA without antibody or morpholino)

Despite partial removal of ZP2 by *Trim-Away*, the functional consequences were overt. ZP2 KD embryos were retarded on day 2 (on average 15.0% had reached the 8-cell stage at the 44 h scoring compared with 41.6% of paired controls; Supplementary Figure S6) and day 3, and the morulae struggled to form blastocysts, even though in the end they caught up and succeeded with similar blastocyst rates compared to mock KD and MC groups (Fig. 5A; Supplementary Table S4). Cavitation was scored once daily rather than continuously, so the delay could not be resolved more finely than the scoring interval; yet the effect of retardation was evident from the total cell numbers at the blastocyst stage: MC, 41.7 ± 12.1 cells (*n* = 21 blastocysts); mock KD, 42.5 ± 13.5 cells (*n* = 64 blastocysts); ZP2 KD, 26.8 ± 8.1 cells (*n* = 33 blastocysts) (*p* < 0.0001, Wilcoxon). The total cell number reduction in ZP2 KD blastocysts (≈ 36%) was not distributed uniformly across cell lineages, as revealed by IF analysis of epiblast (NANOG+), primitive endoderm (SOX17+) and trophectoderm (CDX2+): NANOG+ epiblast cells were 5.1 ± 3.6 in ZP2 KD versus 5.3 ± 2.3 in mock KD (*p* = 0.3694, Wilcoxon), i.e. preserved in absolute terms; CDX2 + trophectoderm cells fell from 33.9 ± 10.9 in mock KD to 20.3 ± 6.2 in ZP2 KD (a 40% reduction, *p* < 0.0001, Wilcoxon); SOX17 + primitive endoderm cells fell from 3.3 ± 2.5 in mock KD to 1.5 ± 1.5 in ZP2 KD (a 55% reduction, *p* = 0.0002, Wilcoxon) (Fig. 5B; Supplementary Figure S7; Supplementary Table S5). To explore if the deficits in trophectoderm and primitive endoderm were transients or were compensated for at a later stage, ZP2 KD blastocysts were tested for implantation competence in vitro (outgrowth assay) and postimplantation development in vivo (embryo transfer). Compared to controls, ZP2 KD blastocysts had a significantly reduced ability to hatch from the zona pellucida and attach to the feeder layer of fibroblasts in the outgrowth assay in vitro (*p* = 0.001, ZP2 KD vs. MC, Fisher exact test), consistent with reduced CDX2 + cell numbers (Fig. 5C; Supplementary Table S6). In vivo, the proportion of transferred blastocysts that implanted was similar across the groups (49% for ZP2 KD, 41% for mock KD, 45% for MC), whereas the proportion of implantation sites yielding a fetus was lower after ZP2 KD (43%, 16/37) than after mock KD (63%, 10/16) or MC (79%, 15/19) (*p* = 0.039, ZP2 KD vs. MC, Fisher exact test) (Fig. 5C; Supplementary Table S7).

### Proteomic and transcriptomic correlates of unhealthy blastocyst formation after KD of ZP2

In search of mechanistic correlates for the reduced ability of ZP2 KD mouse embryos to form healthy blastocysts in a timely fashion, relevant developmental stages were subjected to transcriptome and proteome analysis. The transcriptome and proteome were collected at different stages deliberately, because in a *Trim-Away* experiment, the direct effect is on the proteome and transcriptomic effects are subsequent. Consistent with this temporal logic, the proteome was analyzed at the time of phenotypic manifestation (delayed morula-blastocyst transition), while the transcriptome was analyzed at the blastocyst stage.

**Fig. 6:**
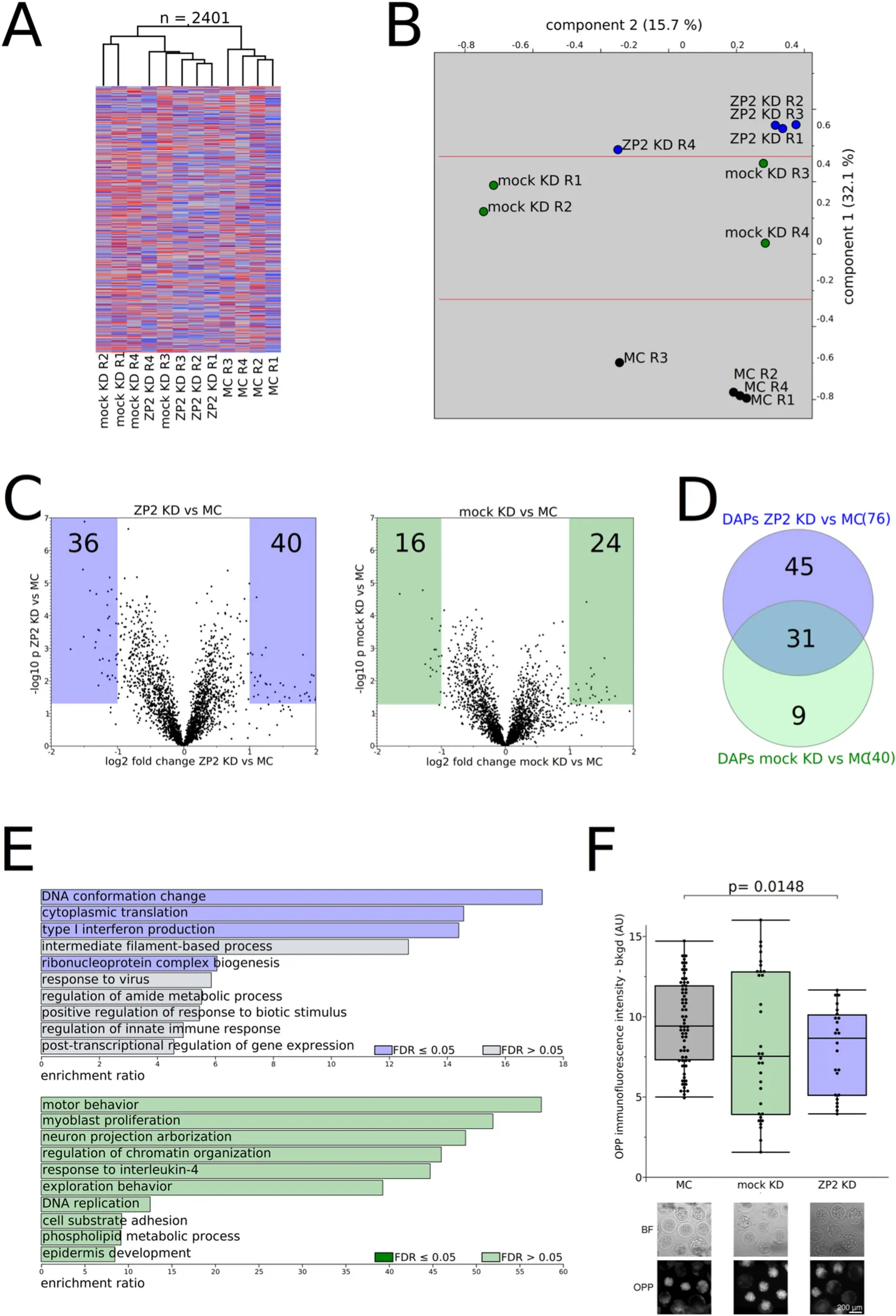
Proteomic correlates of unhealthy blastocyst formation after KD of ZP2. Zygotes were microinjected with *Trim-Away* reagents and cultured to later morula stage. **A** Hierarchical clustering of the 2401 proteins detected in all samples (listed in Supplementary Table S9). **B** Principal component analysis of the samples based on the 2401 proteins. **C** Volcano plots of the proteins differentially expressed between ZP2 KD and MC and between mock KD and MC, highlighting the proteins that satisfy the criteria of both *p* < 0.05 (Student’s t-test) and |fold change|>2. The numbers in the colored areas indicate the respective DAPs. **D** Venn diagram identification of DAPs in ZP2 KD versus mock KD. Gene lists corresponding to the DAPs are provided in Supplementary Table S10. **E** Over-representation analysis of the DAPs between ZP2 KD (top) and mock KD (bottom) in the ontology “Biological process”. **F** Quantification of protein synthesis by OPP assay, with representative images. P-values in (**F**) were calculated with Wilcoxon test. Abbreviations: DAP, differentially abundant protein; ZP2 KD, ZP2 knockdown by *Trim-Away* ZP2 + morpholino anti-*Zp2*; mock KD, mock knockdown by *Trim-Away* GFP + morpholino anti-*Gfp;* MC, micromanipulation control (overexpression of *mCherry-Trim21* mRNA without antibody or morpholino); OPP, O-propargyl-puromycin

For proteome analysis 30 embryos were collected as late morulae in quadruplicate (R1, R2, R3, R4) for each of the treatments (ZP2 KD; mock KD; MC), deprived of the external zona and subjected to mass spectrometric analysis in data-independent acquisition (DIA) mode (see Methods). The full dataset has been deposited in PRIDE (PXD062983). Overall, the dataset had a depth of 4531 proteins (Supplementary Table S8), of which 2401 were present in all samples in all replicates (Supplementary Table S9). The 2401 proteins dataset was used for further analysis. The samples clustered according to treatment (Fig. 6A). Principal component analysis separated the ZP2 KD from the other two treatments in the 1st component (32.1% of the variance; 2nd component, 15.7%; Fig. 6B). To isolate ZP2-specific effects, a two-step procedure was followed (see also Materials and Methods). First, Volcano plots were applied to identify the proteins that are perturbed in the ZP2 KD or mock KD relative to MC (t-test, p < 0.05; |fold change|>2; Fig. 6C). Then, these two sets of perturbed proteins were subjected to Venn diagram intersection (Fig. 6D). The resulting two unique subsets (Supplementary Table S10) were interrogated against the genome to perform over-representation analysis for ‘Biological Process’ using the WEB-based GEne SeT AnaLysis Toolkit (WebGestalt; see Methods). This analysis revealed that four of the top 10 annotated terms in ZP2 KD are significant (FDR < 0.05), while none of the top 10 terms is significant in mock KD (Fig. 6E). The enrichment of ‘type I interferon production’ in the ZP2 KD-exclusive set indicates that TRIM21 is more strongly engaged in ZP2 KD than in mock KD (more complexes between antibody and protein of interest form). This is expected from the activation versus mere presence of TRIM21, which is an innate immune sensor that, when activated, can trigger NF-κB and interferon signaling pathways [49]. Two other GO terms are related to protein synthesis. These two terms resonate with previous study of this group on ZP3, in which the knockdown of ZP3 reduced protein synthesis in the 2-cell mouse embryo [15]. Therefore, this line of inquiry was followed also in the present study, using an orthogonal method to test whether protein synthesis was affected. To this end, the global protein synthesis of morulae using the OPP assay, as described (see Methods). ZP2 KD embryos exhibited lower OPP values, and this reduction was significant compared to MC (Fig. 6F). The results of the OPP assay support the main finding of the GO analysis conducted in the proteome, namely, that ZP2 KD is associated with reduced global protein synthesis, which may reflect a specific role of ZP2 in supporting translation.

**Fig. 7:**
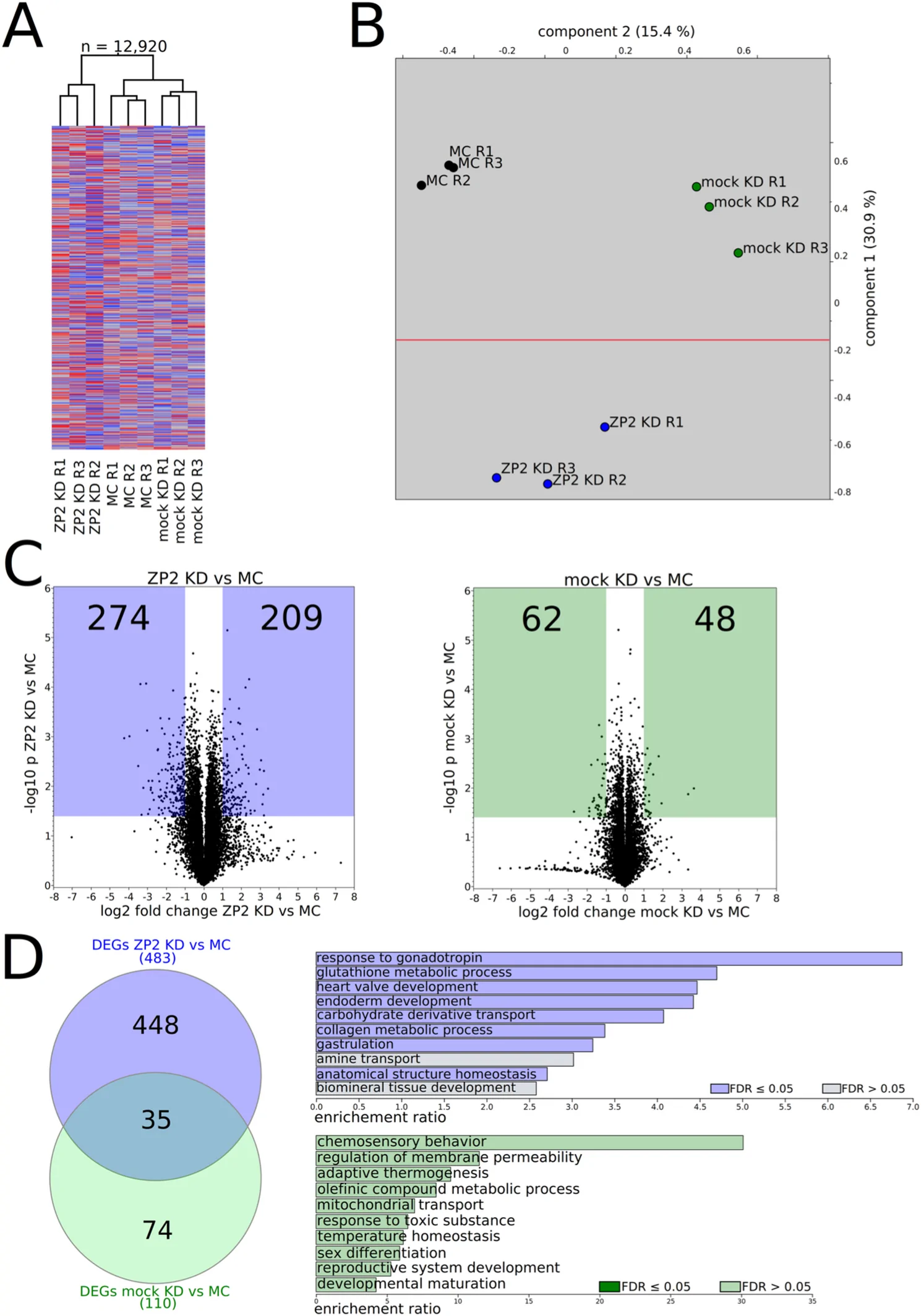
Transcriptomic correlates of unhealthy blastocyst formation after KD of ZP2. Zygotes were microinjected with *Trim-Away* reagents and cultured to blastocyst stage. **A** Hierarchical clustering of the 12920 protein-coding mRNAs detected in all samples (listed in Supplementary Table S13). **B** Principal component analysis of the samples based on the protein-coding mRNAs. **C** Volcano plots of the DEGs between ZP2 KD and MC and between mock KD and MC, highlighting the mRNAs that satisfy the criteria of both *p* < 0.05 (Student’s t-test) and |fold change|>2. The numbers in the colored areas indicate the respective differentially expressed mRNAs. **D** Venn diagram identification of DEGs in ZP2 KD versus mock KD. Gene lists are provided in Supplementary Table S10. Over-representation analysis of the DEGs between ZP2 KD (top) and mock KD (bottom) in the ontology “Biological process”. Abbreviations: DEG, differentially expressed gene (mRNA); ZP2 KD, ZP2 knockdown by *Trim-Away* ZP2 + morpholino anti-*Zp2*; mock KD, mock knockdown by *Trim-Away* GFP + morpholino anti-*Gfp;* MC, micromanipulation control (overexpression of *mCherry-Trim21* mRNA without antibody or morpholino)

For transcriptome analysis 10 blastocysts were collected in triplicate (R1, R2, R3) for each of the treatments (ZP2 KD; mock KD; MC), and subjected to RNA sequencing to measure the global gene expression (see Methods). The dataset attained a depth of 37740 total RNAs (miRNA, snRNA, snoRNA, rRNA, lncRNA, protein-coding; Supplementary Table S11), of which 17341 RNAs were present in all samples in all replicates (Supplementary Table S12), and 12920 RNAs were protein-coding (Supplementary Table S13). The full dataset has been deposited in ArrayExpress (E-MTAB-16339). The subset of 12920 mRNAs was used for further analysis. Hierarchical clustering showed that the samples clustered according to treatment (Fig. 7A). Principal component analysis separated the ZP2 KD from the other two treatments in the 1st component (30.9% of the variance; 2nd component, 15.4%; Fig. 7B). Adopting the same workflow used for the proteins, Volcano plots were applied to identify transcripts that are perturbed in the ZP2 KD or mock KD relative to MC (t-test, *p* < 0.05; |fold change|>2; Fig. 7C); and Venn diagram intersections were applied to isolate the transcripts that are affected exclusively in the ZP2 KD or exclusively in the mock KD (Fig. 7D). These two subsets (Supplementary Table S10) were subjected to over-representation analysis, as described. This analysis revealed that the top 10 terms in ZP2 KD are all significant (FDR < 0.05) and include annotated terms that are relevant to early postimplantation development (‘endoderm development’, ‘gastrulation’), while none of the top 10 terms are significant in the case of mock KD (Fig. 7D). The GO terms “endoderm development” and “gastrulation” provide a coherent molecular correlate of the phenotype described in Fig. 5.

A legitimate objection is whether the above changes in the proteome and transcriptome are a cause or a consequence, that is: given that blastocysts had less trophectoderm and less primitive endoderm, could the changes in gene expression be a consequence rather than a cause, and could it all simply be a matter of stress caused by the microinjections? This is deemed possible, but unlikely, given the following. At the morula stage, before the trophectoderm and primitive endoderm deficits became manifest, ZP2-depleted embryos already showed a specific reduction in translational capacity that cannot be attributed to altered cell lineage composition. Consistent with a translational deficit coming first, the transcriptome of ZP2 KD blastocysts showed a coordinated elevation of the translational apparatus (251 of 287 detected genes went up in the groups of cytoplasmic ribosomal proteins, mitochondrial ribosomal proteins, ribosome-biogenesis factors, translation-initiation factors; Supplementary Table S14). On the contrary, at the protein level in morulae, the gene set of the translational apparatus moved in the opposite direction (96 of 128 detected proteins went down; Supplementary Table S14). The opposite directions of these two observations - ribosomal protein content reduced at the morula stage, ribosomal transcripts elevated at the blastocyst stage - is what would be expected if the embryo were compensating for a shortfall in translational capacity. As for cellular stress, relevant terms such as chaperone, unfolded-protein response, ubiquitin-conjugating enzymes and proteasome subunits were not over-represented in the ZP2 KD proteome, even though some of them were present in the transcriptome (Supplementary Table S14).

## Discussion

The main finding reported here seems to go against a body of scientific literature: there is an abundant intracellular fraction of ZP2 that contributes to the timing of the morula-to-blastocyst transition and to blastocyst quality in mice. In fact, this is not the first time that the literature hinted at embryonic roles of ZP proteins, at variance with the canonical model of the zona serving only as extracellular matrix which the oocyte passively transmits to the embryo (Introduction). Prompted by past discrepancies in the ZP2 narrative, we tested whether the ZP2 present within the embryos served any function, by removing it. We emphasize the distinction between proteasomal removal of the intracellular ZP2 (*Trim-Away* in this study) and removal of the outer zona using Tyrode’s solution by other investigators [50]. When embryos were cultured without the outer zona (zona-free), there were repercussions on gene expression [50], but this is not how experiments were conducted in this study: the embryos were zona-enclosed until sampling and processing, and our interest was directed to the role – if any – of the intracellular ZP2. To guide a discussion of the discrepancy between our findings and the canonical model of the zona, we are going to formulate three specific questions about our findings. First finding was that stripping mouse embryos of the zona pellucida revealed an intracellular fraction of ZP proteins that endured preimplantation development. Therefore, how to explain that we detected ZP proteins and particularly ZP2 inside mouse embryos when previous investigators did not? Second finding was that protein KD of ZP2 impeded healthy blastocyst formation. Therefore, how to explain that the ZP2 KD embryos in this study suffered reduced postimplantation rates yet survived, whereas embryos of *Zp2*-null oocytes had died [6]? Third and last finding was about the molecular correlates of unhealthy blastocyst formation after KD of ZP2. Therefore, what is the function of intracellular ZP2 protein? After answering these three questions, we will conclude by prompting a reflection on the definition of oocyte-specific genes - the family to which the *Zp* genes have traditionally been assigned.

The first question is how to explain that we detected ZP proteins and particularly ZP2 inside mouse embryos when previous investigators did not, and that ZP2 even seemed to increase. We think the reason for detection vs. previous non-detection is technical, not biological. Since the zona is a secreted matrix that forms a coat around the cell, in the past it seemed nonsensical to remove the very thing that one wanted to detect, and so the zona was left in place in IF studies of preimplantation embryos. This way, however, the coat acted like a sponge that bound the antibody before this could penetrate deeper, ultimately obscuring the intracellular deposit of ZP proteins. An analogous artefact has been described for indirect IF of oocyte‘s subcortical maternal complex components, where peripheral structures can trap antibodies before deeper penetration is achieved [51]. We hypothesize, by extension, that the much thicker zona could exert a similar masking effect. A parallel gap exists in the immunoblotting literature: ZP2 has been immunoblotted in mouse oocytes, zygotes and 2-cell embryos, where the readout of interest was cleavage by ovastacin at fertilization, but not across later cleavage stages [52–54] and not in zona-free material in which the extracellular coat no longer dominates the signal. The intracellular fraction was therefore outside the sampling frame of both classes of experiment. Returning to immunofluorescence, post-fixation removal of the zona preserved intracellular antigen distribution while eliminating the strong extracellular signal that otherwise would have dominated the image. This way, intracellular ZP2 was exposed by both direct and indirect ZP2 IF using a monoclonal antibody of proven specificity. In addition to being intracellular, ZP2 even seemed to increase, as supported by two methods (MS, IF). It is tempting to think that ZP2 *de novo* synthesis occurred from mRNA, but we are cautious about proposing this. While there are indeed *Zp/ZP* mRNAs bound to ribosomes (as shown by translatome studies in both mice [7, 8] and humans [37]), the decisive experiment would be a stable isotope labeling with amino acids in cell culture (SILAC) [55]. So far, in preliminary studies, we have not been able to demonstrate significant incorporation of isotopic amino acids into ZP proteins during mouse embryo culture in vitro, but this might be a technical glitch in our experiments, as there is an independent study showing that the *Zp3* gene promoter is active in mouse blastocysts (Cre recombinase encoded by a paternal *Zp3*–Cre knock-in allele caused reporter activation in blastocysts containing a floxed tdTomato reporter [56]). In addition to being intracellular, part of ZP2 appeared to reside outside the cis-Golgi and the ER. One decisive observation is consistent with this non-Golgi, non-ER fraction being accessible from the cytosol: its susceptibility to *Trim-Away*, a method that depletes only antibody-accessible protein because microinjected antibodies do not cross the membranes of intact secretory compartments. It should be noted, however, that these observations do not formally establish free cytosolic residence: our basic colocalization microscopy can exclude some compartments but cannot prove the absence of association with compartments for which no marker was tested, and the partial (~ 50%) *Trim-Away* depletion indicates only that part of the total ZP2 pool is antibody-accessible. Definitive assignment of ZP2 would require selective permeabilization, which already supported the unusual subcellular localization of ZP3 [15], or electron microscopy analyses, which is beyond the scope of this study. Until then, the cytosolic assignment of a fraction of ZP2 is a hypothesis. With regard to how proteins of the secretory pathway can be found outside it, we refer to supporting evidence in the literature. It has been shown that compartmentalization in the secretory pathway is leaky, i.e., some proteins fail to stay compartmentalized due to retro-translocation from ER and Golgi back in the cytosol [57, 58], or fail to translocate in the ER in the first place, due to malfunction in the N-terminal signal peptide [59] or absence of the signal peptide. It was shown in case of ZP3 that there is a variant lacking the signal peptide, resulting in internal localization within cancer cells, which contrasts with the canonical extracellular role of ZP3 in oocytes [60].

The second question is how to explain that some of our ZP2 KD embryos developed to late gestation (recovered at E16.5) after transfer, whereas no live births were ever reported from blastocysts derived from *Zp2*-null oocytes [6]. It should be noted that in a previous study of ours, a pilot KD experiment of ZP2 resulted in a more severe and earlier-onset phenotype (arrest at the 2–4-cell stage [15]) than in the present study (delay of the morula-to-blastocyst transition). One difference between these studies lies in the antibodies used. In this study, the anti-ZP2 antibody was found to be superior to that in the previous study. Back to the question of how to explain the phenotypic discrepancy, two non-mutually-exclusive explanations are plausible: (1) *Zp2*-null oocytes were of lower quality, as the original Authors proposed [6]; (2) our *Trim-Away*-mediated depletion was only ≈ 50% efficient, possibly not reaching the threshold for total embryonic lethality. As regards the oocyte quality, the Authors of the original *Zp2*-null oocytes study noted that “in addition to its role in fertilization and protection of early embryos, these data are consistent with the zona pellucida maintaining interactions between granulosa cells and oocytes during folliculogenesis”. Therefore, it could be that the embryos from *Zp2-*null oocytes did not develop to term because the oocytes were defective. However, other studies reported developmental abnormalities also in wildtype oocytes that had already completed maturation before the zona was removed [15, 50, 61], thereby suggesting embryonic roles of ZP proteins that are distinct from the oocytic roles. As regards the limited extent of ZP2 KD, the ≈ 50% depletion should be viewed in the context of a protein of interest that is highly abundant and partly shielded inside secretory vesicles, whereby it is impossible to remove all of it, even after pushing *Trim-Away* to the boundaries of its technical possibilities (use of maximum reagent concentrations before TRIM21 becomes toxic [47]). This stoichiometric consideration aligns with one of the remarks, in the original *Trim-Away* publication [13], that “TRIM21 and antibody levels could become limiting during *Trim-Away* if the target is in significant molar excess.” This consideration has now been substantiated by us: upon re-analysis of our independent proteomic dataset [62], the molar abundance of ZP2 (riBAQ) turned out to exceed that of the microinjected antibody (IgG), of TRIM21, and of the CHERRY tag of TRIM21, placing the substrate in molar excess over the depletion machinery and thereby imposing a stoichiometric ceiling that is consistent with the ≈ 50% plateau (Supplementary Figure S8). A KD does not necessarily have to be 100% to be phenotypically consequential: overt phenotypes were observed after extents of KD as low as 16% [63, 64]. Since the extent of KD for ZP2 was partial, it would be futile to conduct a rescue experiment, for instance by overexpressing ZP2 protein from a morpholino-resistant mRNA in the background of ZP2 KD. This would be tantamount to adding protein on top of an already highly abundant amount of the same. It should be noted that *Trim-Away* necessarily imposes a proteasomal load through TRIM21-mediated degradation of the protein of interest, and because ZP2 is far more abundant than almost any other protein, the ZP2 knockdown engages TRIM21 more strongly, as reflected in the enrichment of the type I interferon production term in the ZP2-knockdown-exclusive set. Two observations nonetheless argue against a generic proteotoxic-stress explanation of the ZP2 KD phenotype, namely: (1) the affected gene-ontology terms are specific (cytoplasmic translation in the proteome; endoderm development and gastrulation in the transcriptome) rather than the chaperone or unfolded-protein signature expected of proteotoxic stress, and (2) overloading the proteasome with ZP2 would be tantamount to limiting the proteome’s ability to degrade other maternal proteins during the oocyte-to-embryo transition, which has been shown to cause early cleavage arrest [65], whereas the ZP2 KD embryos progressed to morula and blastocyst. The affected gene-ontology terms are specific (cytoplasmic translation in the proteome; endoderm development and gastrulation in the transcriptome) rather than the chaperone or unfolded-protein signature expected of proteotoxic stress (Results).

The third question is about the function of intracellular fraction of ZP2 protein. Our transcriptomic data revealed that the functions affected by the ZP2 KD are consistent with the observed phenotype, namely disturbance of the morula-blastocyst transition. However, there are quite distinct gene products that come to mind when one thinks of the morula-blastocyst transition (*Sf3b14*, *Sf3b1*,* Rpl7l1*, and *Rrp7a* [66]; *Wdr74* [67]; *ZO-1 a.k.a. Tjp1* [68]; *Gm11545* [69]; *Tcfap2c* [70]; *Cdx2* [71]; *Cdh1* [72]; *Exosc10/Rrp6* [73]; *Dcaf13* [74], *Traube (Trb)* a.k.a. *Aatf* [75]; *Actl6a*, *Gabpa*, *Hist1h3*, *Matr3*, *Mfng*, *Mxi1*, *Nop2*, *Pbrm1*, *Pnldc1*, *Ptpn18*, *Rpl7l1*, *Rrp7a*, *Rtn4*, *Sf3b1*, *Sf3b6*, *Supt6*, *Tm4sf1*, *Txnrd3*, *Uspl1*, and *Wdr74* [76]). Although adding *Zp2* to this list may seem unwarranted given its canonical oocyte-specific extracellular identity, precedent exists for genes acquiring unrecognized developmental roles. *Nr5a2*, for example, was known for its role in embryonic genome activation at the 2-cell stage in mice [77], and was not thought to play a role in the morula-blastocyst transition, until it was shown that it did [78]. An alternative possibility is that reduced ZP2 affects lineage allocation indirectly, through the chromatin context in which *Cdx2* and *Sox17* are activated. Trophectoderm and primitive endoderm specification depend on timely establishment of H3K4me3 and H3K9me3 at lineage loci [79, 80], and a delayed morula-to-blastocyst transition could displace that timing without ZP2 acting on the loci directly. Two readings of this are possible. The first is that reduced translational output at the morula stage is a downstream consequence of a generally perturbed embryo, in which case the enriched translation terms are a symptom rather than a mechanism. The second, which we adopt as a working hypothesis rather than as a demonstrated mechanism, is that intracellular ZP2 contributes directly to sustaining translation. We favor the second as the more parsimonious reading, because the proteome was sampled at the onset of the phenotype (late morula) whereas the transcriptomic changes were read at the blastocyst stage, and because an equivalent translational deficit followed the knockdown of the related protein ZP3 [16]. Moreover, it has been shown KD of ribosomal protein genes affected embryonic cell numbers and morula-blastocyst transition [44]. Nonetheless, a physical link between ZP2 and the translation machinery is not established yet. Testing it would require immunoprecipitation, with a cautionary note: fact that proteins interact with each other in a cell lysate - where everything is artificially mixed together - does not mean that those same proteins also interact under physiological conditions. To date, 19 candidate binding partners are proposed for human ZP2 (UBR3, AMY1C, C6ORF58, CST1, CST4, EXT1, FAR2, HPCAL1, IGHA1, IGHA2, IGLC7, INTS7, MUC7, PIGR, RHOBTB3, SLC25A4, TSC2, YWHAZ, ZG16B), as revealed by a query made with BioGRID [39] (accessed in March 2026). These interactors are mostly from non-embryonic contexts and include TSC2, which has established roles in mTOR signaling — a pathway highly relevant to blastocyst physiology and metabolism [81].

While this study provides important insights, several limitations should be acknowledged. First, the intracellular ZP2 pool was defined operationally, by exclusion: it is the fraction that remained after zona removal with Tyrode’s solution, that was accessible to a microinjected antibody, and that did not overlap the cis-Golgi marker GM130 or the ER marker Calreticulin. Free cytosolic residence is therefore a hypothesis rather than a demonstrated fact. Second, the depletion achieved was partial (≈ 50%) and is bounded by the molar excess of ZP2 over the depletion machinery (Supplementary Figure S8), so the phenotype reported here is that of ZP2 reduction and not of ZP2 loss; no rescue experiment was performed, for the stoichiometric reasons set out above. Third, ZP2 was detected and depleted with a single validated monoclonal antibody (IE-3), and no second independent antibody, peptide-competition control or *Zp2*-null material was available to us. Fourth, the apparent stage-specific increase in ZP2 rests on relative quantification (riBAQ, IF integrated density) rather than on absolute amounts, and *de novo* embryonic synthesis of ZP2 remains unproven: the ribosome-profiling reanalysis places translational engagement of *Zp2* in the oocyte-to-4-cell window, and the protein present at later stages may therefore be persisting maternal protein. Fifth, the link between intracellular ZP2 and translation is a working hypothesis: no physical association with ribosomes or translation factors has been demonstrated, and reduced OPP incorporation is compatible both with a direct contribution of ZP2 to translation and with translation being an early downstream consequence of a perturbed morula. These limitations do not diminish the finding of this study (Conclusions), but they suggest avenues for future research: it will be sensible to perform (1) ZP2 immunoprecipitation to identify its interaction partners, (2) immunogold electron microscopy to precisely determine the subcellular localization of ZP2, and (3) metabolic labelling - for example, using SILAC - to clarify the origin of the apparent increase in ZP2 at the 8-cell/morula stage. However, the most important undertaking of all will be to examine human embryos for the presence of intracellular ZP proteins.

## Conclusions

Bearing in mind the limitations, two conclusions follow from this study. First, ZP2 is present inside mouse preimplantation embryos in a pool that is distinct from the extracellular zona, and partial depletion of that pool delays the morula-to-blastocyst transition and reduces trophectoderm and primitive endoderm allocation. The intracellular pool is therefore functionally relevant, although the depletion achieved was partial and the phenotype should be read as impaired timing and quality of blastocyst formation rather than as an absolute requirement for it. Second, designation of a gene as oocyte-specific should be reconsidered keeping in mind that it describes where the gene was transcribed, not necessarily where its product acts: once deposited in the oocyte, ZP2 performs functions that are not confined to the oocyte. Whether an equivalent intracellular pool exists in human embryos remains to be established, since the available human embryo proteomic data derived from zona-intact material in which the coat masks any intracellular contribution.

## Supplementary information

Below is the link to the electronic supplementary material.

Supplementary Material 1

## Data Availability

All data supporting the findings of this study are available within the article and the accompanying supplementary information files. Large-scale data (omics) have been deposited in public repositories. The mass spectrometry dataset of the developmental series presented in Fig. 1 has been deposited in the PRIDE repository [82, 83] with accession number PXD056725 [84]. For convenience this dataset is also available in processed form as Supplementary Table S1. The normalized integrated densities of ZP2 IF intensities measured in preimplantation embryos and presented in Fig. 3 are available in Supplementary Table S2. The data of the translational inhibition of *Zp2* mRNA presented in Supplementary Figure S4 are available in Supplementary Table S3. The raw data of preimplantation development, blastocyst quality and postimplantation development after ZP2 KD presented in Fig. 5 are detailed in Supplementary Tables S4-S7. The mass spectrometry dataset of Fig. 6 has been deposited in the PRIDE repository with accession number PXD062983 [85]. For convenience this dataset is also available in processed form as Supplementary Tables S8-S9. The differentially expressed proteins or transcripts in ZP2 KD, mock KD, and MC are presented in Supplementary Table S10. The RNA-seq dataset of Fig. 7 has been deposited in the ArrayExpress with accession number E-MTAB-16339 [86]. For convenience this dataset is also available in processed form as Supplementary Tables S11-S13. The analysis of ribosomal and stress control gene sets among the differentially abundant proteins or transcripts is presented in Supplementary Table S14. All supplementary Tables are deposited in FigShare [87].

## Abbreviations

ARRIVE: Animal Research: Reporting of In Vivo Experiments
BP: biological process
BSA: bovine serum albumin
DAP: differentially abundant protein
DDA: data-dependent acquisition
DEG: differentially expressed gene (mRNA)
DIA: data-independent acquisition
eCG: equine chorionic gonadotrophin (pregnant mare’s serum gonadotrophin)
FDR: false discovery rate
FPKM: fragments per kilobase of exon per million mapped fragments
GFP: green fluorescent protein
GO: gene ontology
hCG: human chorionic gonadotrophin
HCZB: Hepes-buffered Chatot, Ziomek and Bavister medium
iBAQ: intensity-based absolute quantification
IF: immunofluorescence
KD: knockdown
KSOM(aa): potassium simplex optimization medium with amino acids
LC-MS/MS: liquid chromatography with tandem mass spectrometry
MC: micromanipulation control
MS: mass spectrometry
OGDB: Oregon Green dextran beads
OPP: O-propargyl-puromycin
PCA: principal component analysis
PrE: primitive endoderm
riBAQ: relative iBAQ
RNA-seq: RNA sequencing
SILAC: stable isotope labeling with amino acids in cell culture
TE: trophectoderm
ZP: zona pellucida
ZP1, ZP2, ZP3, ZP4: zona pellucida proteins 1 to 4
