# Supplementary material for: Proteomic and transcriptomic analysis reveals an intracellular role for zona pellucida protein 2 in mouse morula-blastocyst transition
Source: BMC Genomics. 2026 Oct 22;27:885. doi: 10.1186/s12864-026-13487-z (PMC13704890; doi:10.1186/s12864-026-13487-z)
Supplement: Supplementary file 1 [file 12864_2026_13487_MOESM1_ESM.pdf]

Supplementary information

Proteomic and transcriptomic analysis reveals an intracellular role for zona pellucida protein 2 in mouse morula-blastocyst transition

Thomas Nolte, Steffen Israel, Hannes C.A. Drexler, Georg Fuellen, Julina Wiese and Michele Boiani

Supplementary Figures: pages 2-11

Supplementary Tables: repository link and DOI on page 12

References: page 13

Supplementary figures

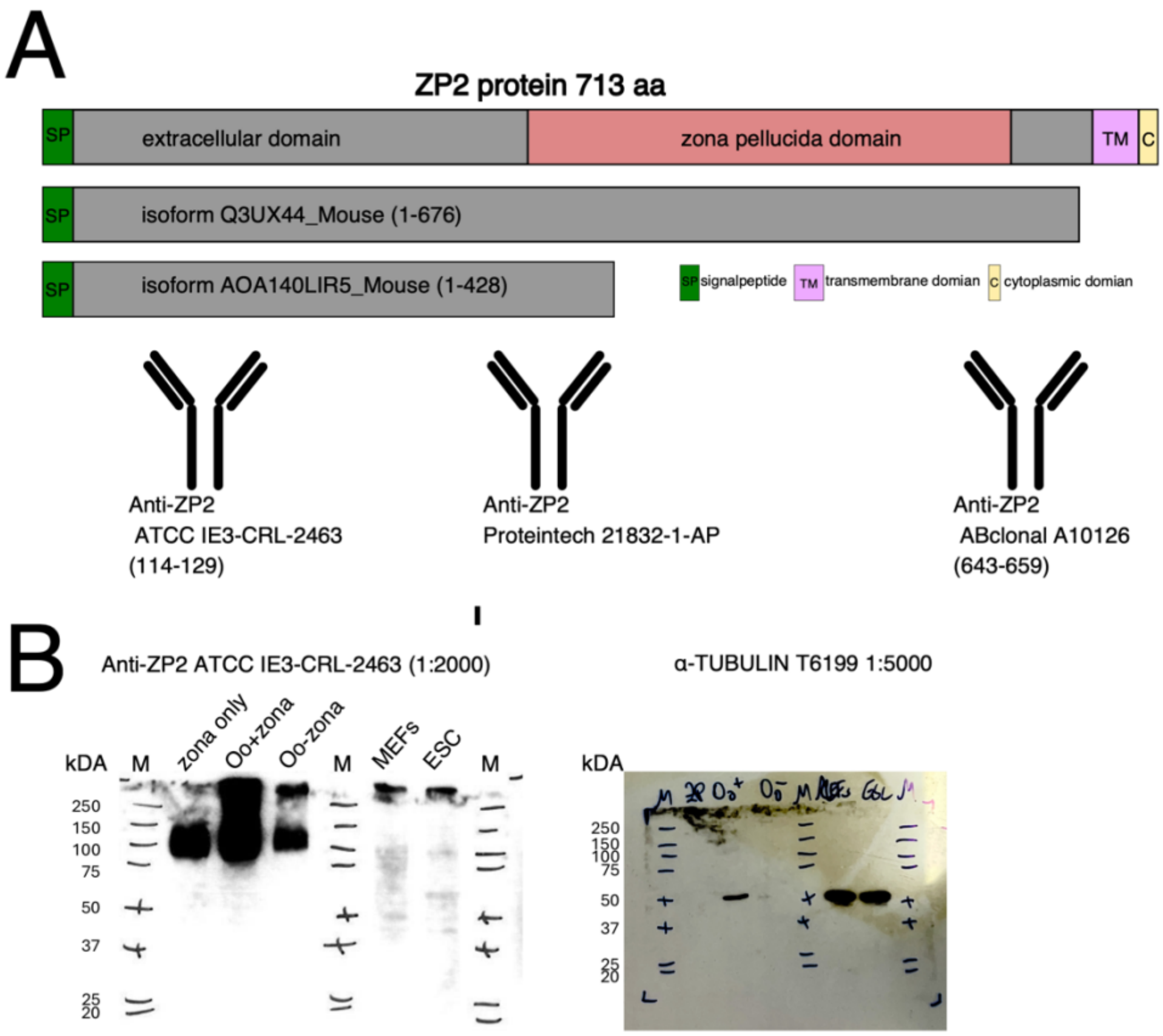

Supplementary Figure S1a. Antibody selection and validation. (A) Schematic of mouse ZP2 protein indicating the epitope binding regions of the three antibodies tested (ATCC IE-3, ABclonal A10126, ProteinTech 21832-1-AP). (B) Immunoblot of oocyte lysate, evacuated zona lysate, zona-free oocyte lysate, MEFs lysate, ESC lysate, processed anti-ZP2 antibody and reprobed for α-TUBULIN as a loading control; only the IE-3 antibody produces a single band of the expected ZP2 molecular weight that is strongest in zona-intact oocyte lysate, weaker in zona-free oocyte lysate and evacuated zona lysate, and with no band at the ZP2 molecular weight in MEF or ESC lysates. Panel B is cropped from the original films, which are presented in full in Supplementary Figure S1b. Protein ladder is Kaleidoscope (Bio-Rad, cat. no. 1610375). Abbreviations: M, molecular size marker; Oo, oocyte; MEFs, mouse embryonic fibroblasts; ESC, embryonic stem cells.

Alt text. Binding sites (A) and specificities (B) of ZP2 antibodies tested in this study. The same blot (B) was probed for ZP2, stripped and reprobed for α-TUBULIN as the loading control, using lysates of oocytes (Oo), mouse embryonic fibroblasts (MEFs) and embryonic stem cells (ESC).

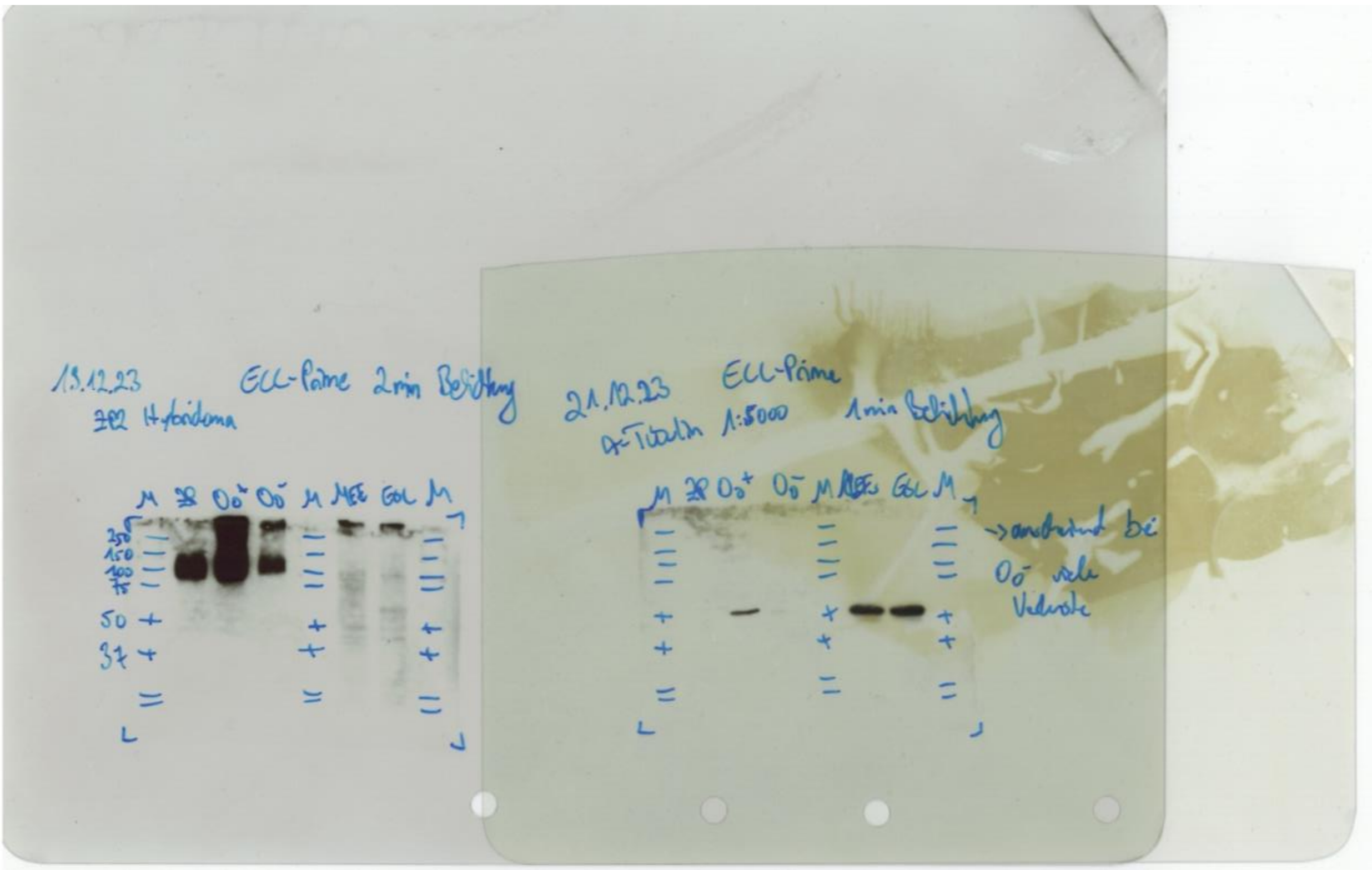

Supplementary Figure S1b. Original AGFA Curix 60 films from which panel B of Supplementary Figure S1a was generated.

Alt text. Two complete films from a single membrane, probed for ZP2 and reprobed for α-TUBULIN, carrying handwritten lane labels and hand-drawn molecular-weight marks. Both films are shown uncropped.

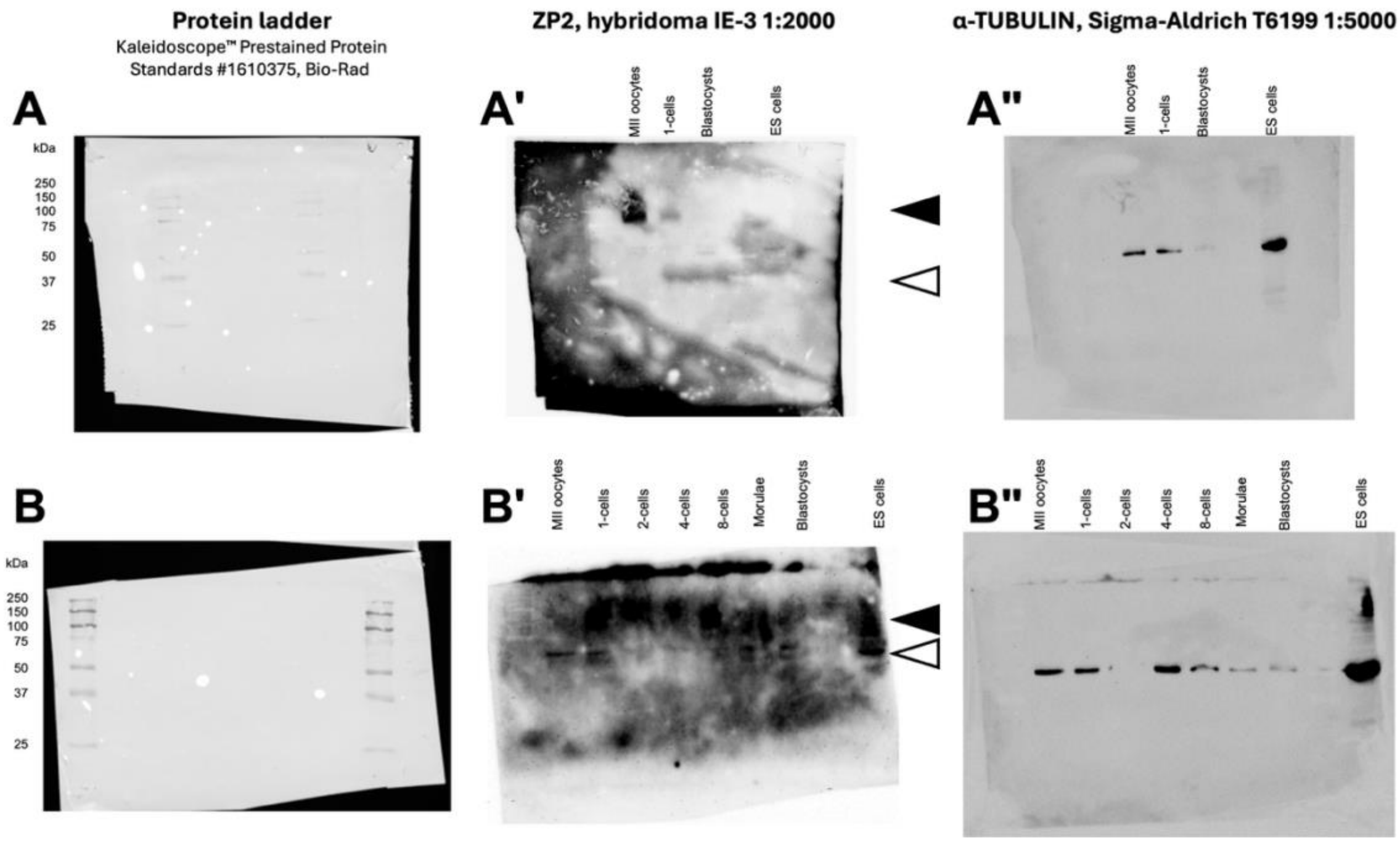

Supplementary Figure S2a. ZP2 protein persists intracellularly in zona-free preimplantation embryos. Immunoblot analysis of pooled oocytes or embryos (n = 100 per lane) probed with the anti-ZP2 monoclonal antibody IE-3 (10 min. exposure), with α-TUBULIN (20 sec. exposure) as loading control, next to the protein ladder (Kaleidoscope, Bio-Rad, cat. no. 1610375). In each row, the first panel is the brightfield image of the protein ladder, the second the ZP2 immunoblot and the third the α-TUBULIN immunoblot. **(A, A', A")** Zona-intact MII oocytes, 1-cell embryos and blastocysts, alongside embryonic stem (ES) cells; the ZP2 signal in these lanes derives from the secreted zona pellucida together with the intracellular protein. IE-3 binds the ZP-N1 domain N-terminal to the ovastacin cleavage site, so both intact ZP2 (≈120 kDa, ◀) and N-terminal cleavage fragment (≈30 kDa, ◁) are detected. **(B, B', B")** Zona-free developmental series - MII oocytes, 1-cell, 2-cell, 4-cell and 8-cell embryos, morulae and blastocysts, in which the zona pellucida was removed before lysis, so that the ZP2 signal reports the intracellular pool alone. The ES-cell lane shows signal at the ZP2 position and its origin was not investigated further. IE-3 binds a product at approximately 120 kDa, consistent with intact glycosylated ZP2 (◀), and a second product at approximately 65 kDa (◁), consistent with a non-glycosylated form of the same backbone. The panels shown here are cropped from the original captures for the sole purpose of separating the two membranes, which were imaged together; each panel corresponds to the whole of the lettered membrane. Uncropped full-length blots are presented in Supplementary Figure S2b.

Alt text. Immunoblots probed for ZP2 with α-TUBULIN as loading control, 100 oocytes or embryos per lane. Panels (A, A', A") show three zona-intact samples: MII oocytes, 1-cell embryos, blastocysts, plus ES cells. Panels (B, B', B") show zona-free samples spanning MII oocytes, 1-cell, 2-cell, 4-cell and 8-cell embryos, morulae, blastocysts, plus ES cells. Molecular-weight markers from 250 to 25 kDa are indicated.

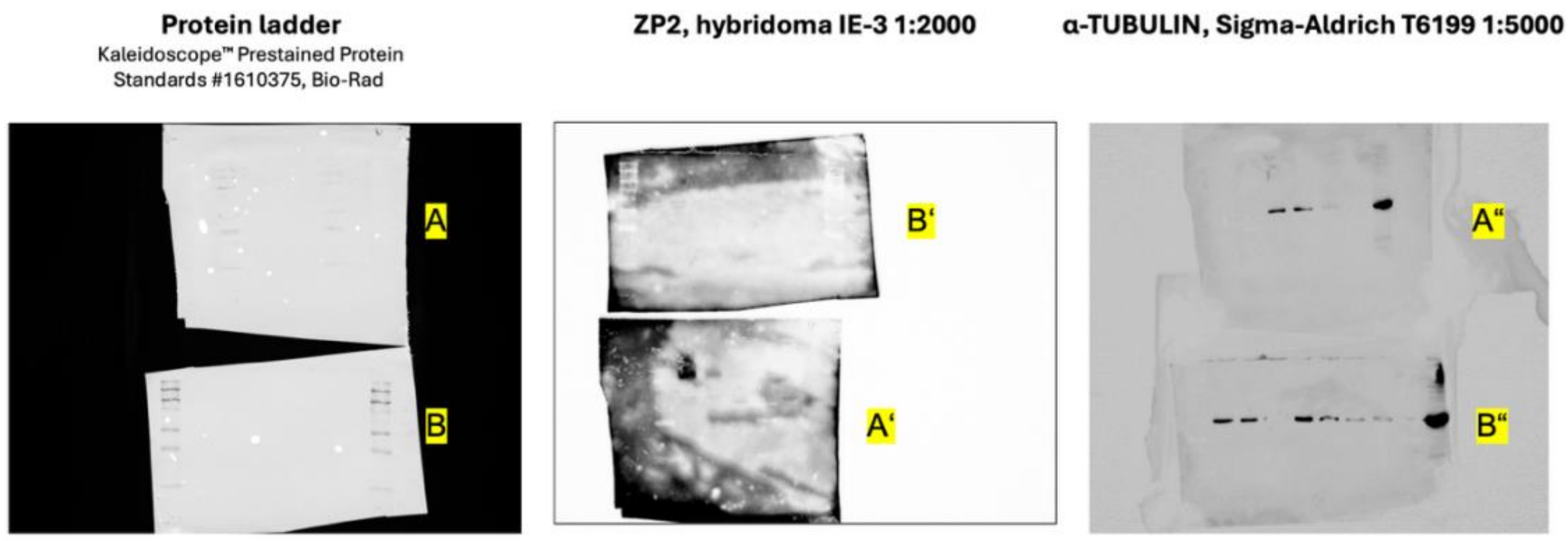

Supplementary Figure S2b. Unmodified immunoblot captures from which the panels of Supplementary Figure S2a were taken. Each membrane is marked with the letter of the corresponding panel in S2a: brightfield protein ladder (A, B), ZP2 (A’, B’) and α-TUBULIN (A”, B”). The zona-intact membrane is A, A’, A”; the zona-removed membrane is B, B’, B”. Note that the two membranes were imaged in opposite vertical order in the ZP2 and the α-TUBULIN captures.

Alt text. Three unmodified ChemiDoc captures, each containing both membranes: white light showing the protein ladder and all membrane edges (A, B), ZP2 (B′ above A′) and α-TUBULIN (A″ above B″). Yellow letters key each membrane to its panel in Supplementary Figure S2a.

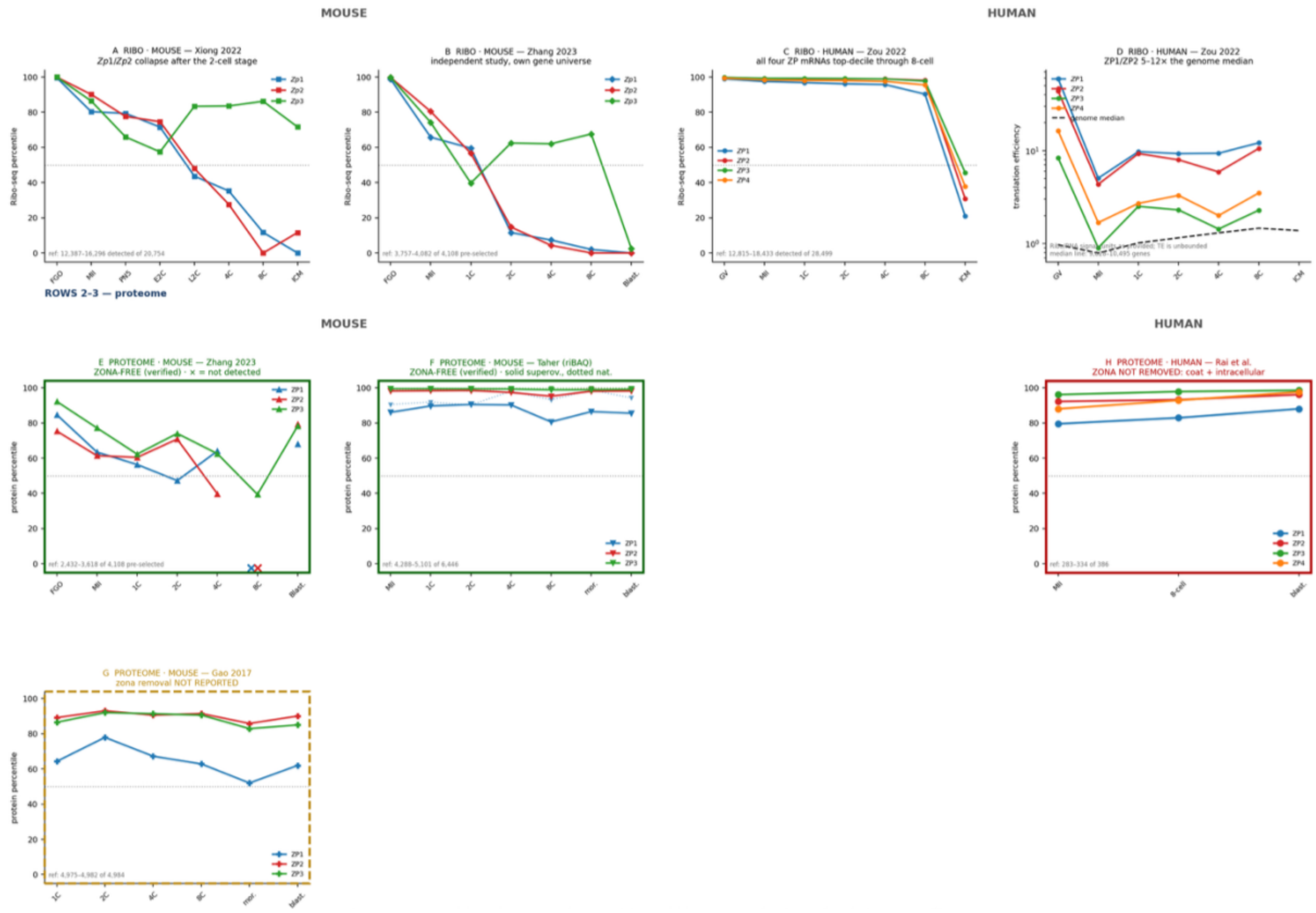

Supplementary Figure S3. Re-analysis of ribosome-profiling, translatome and proteomics datasets of mouse and human embryos [1-6]. First-row ribosome profiling studies show that *Zp* mRNAs are associated with ribosomes up to 2–4 cell stage in mice and up to 4–8 cell stage in humans. Second-row mass spectrometry studies show that ZP proteins are abundantly present in mouse datasets where the outer zona was removed, whilst in humans (where the zona was not removed) little can be concluded. However, the ZP4:ZP2 ratio in the human proteome shifts from 0.65 at MII to 1.29 at blastocyst, suggestive of dynamic events. The third row shows a mass spectrometry dataset of mice, where it is not known whether the zona was removed or not. Regardless of the uncertainties, the common message conveyed by these panels is that intracellular *Zp* mRNAs and intracellular ZP proteins are a likely possibility, but if the outer zona is left in place, then not much can be concluded.

Alt text. Three rows of panels re-analyzing published mouse and human embryo datasets. Row one: ribosome profiling shows *Zp* mRNAs ribosome-associated to the 2–4-cell stage in mouse and 4–8-cell in human. Row two: mass spectrometry shows abundant ZP protein in zona-free mouse samples, but no firm conclusion from zona-intact human samples. Row three: a mouse dataset with unreported zona handling.

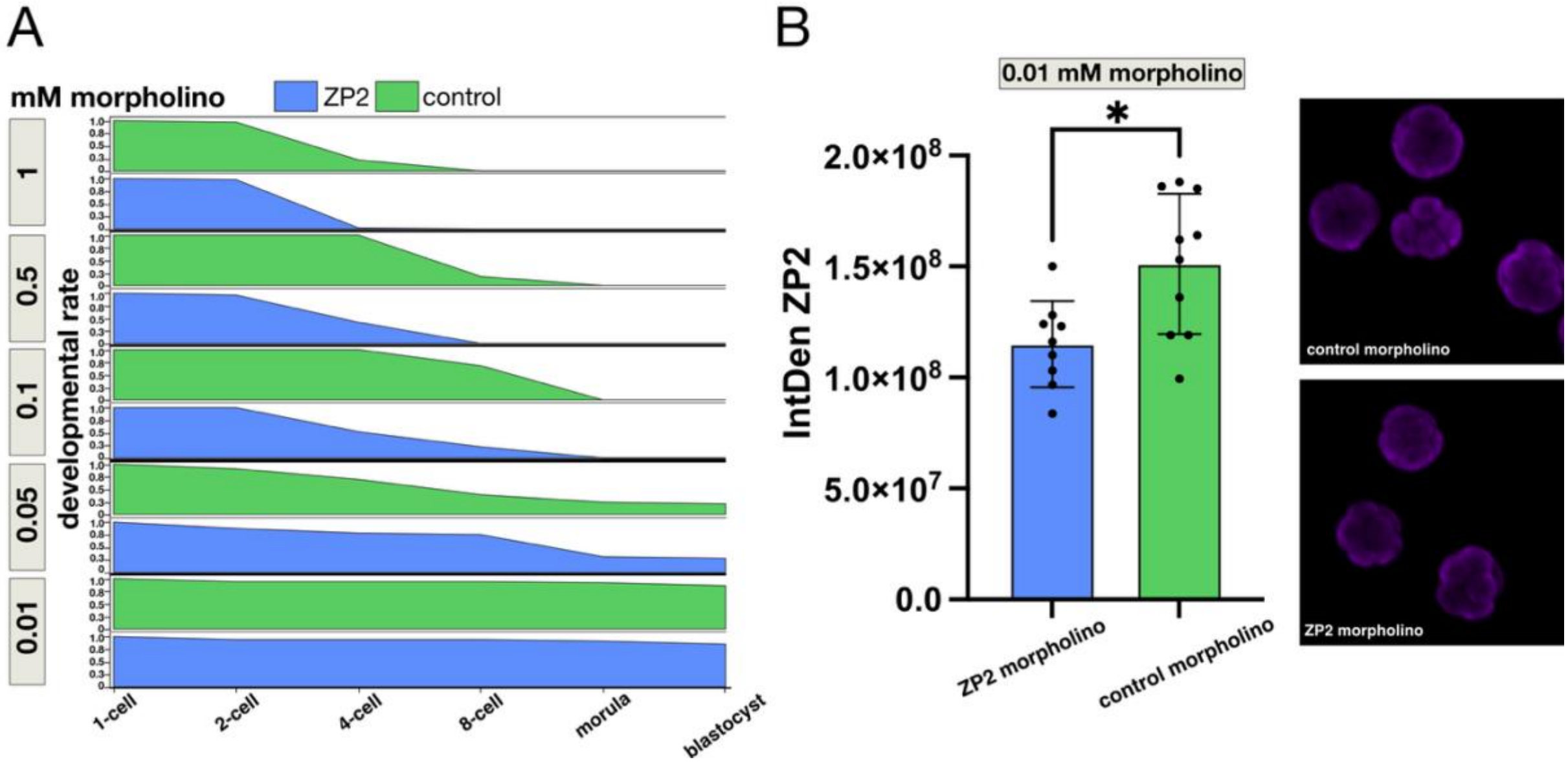

Supplementary Figure S4. Morpholino titration. (A). Developmental progression of zygotes microinjected with various concentrations of morpholino targeting *Zp2* mRNA (treatment) or morpholino targeting an irrelevant sequence (*Gfp*; negative control), using an ICSI needle with an inner diameter of approximately 6-7 µm. This needle delivers greater volumes than those of conventional 1 µm tip microcapillaries. Irrespective of the morpholino target, zygotes can hardly progress to the 8-cell stage at morpholino concentrations higher than 0.1 mM. Although this is suggestive of unspecific toxicity of morpholino no matter which target, it should be noted that targeting *Zp2* is more detrimental than targeting *Gfp*, as seen from the fact that the green bars in (A) are more extended to the right than the blue bars. At morpholino concentration of 0.05 mM both treatment and control zygotes progressed to blastocyst, however at rates substantially lower than 100% also in the negative control, suggestive of unspecific toxicity. At morpholino concentration of 0.01 mM majority of zygotes progressed to blastocyst in both treatment and control groups, indicating that this concentration is suited to test for gene-specific translation-blocking effects without incurring unspecific toxicity. Numbers of zygotes (n) microinjected with anti-*Zp2* morpholino (mM concentration): 34 (0.01), 32 (0.05), 27 (0.1), 24 (0.5), 46 (1.0). Numbers of zygotes microinjected with control morpholino (mM concentration): 36 (0.01), 33 (0.05), 22 (0.1), 22 (0.5), 32 (1.0). (B). Zygotes microinjected with 0.01 mM morpholino were sampled at the 8-cell stage and subjected to ZP2 immunofluorescence using antibody ATCC cat.no. IE-3 – CRL-2463. The immunofluorescence intensity values are provided in Supplementary Table S3. Immunofluorescence intensity quantification revealed that ZP2 intensity was reduced by ~ 20% after translational inhibition of *Zp2* mRNA compared to that of negative control. Representative images of the 8-cell embryos are shown after immunostaining with anti ZP2 (ATCC cat.no. IE-3 – CRL-2463) revealed by secondary antibody. P-value was calculated with Wilcoxon test. *, <0.05. Abbreviations: IntDen, Integrated density of immunofluorescence signal (intensity x area); ICSI, intracytoplasmic sperm injection.

Alt text. Two-part morpholino titration. Panel (A) is a horizontal stacked bar plot showing developmental progression of mouse zygotes microinjected with anti-*Zp2* morpholino or anti-*Gfp* control morpholino at five concentrations (0.01, 0.05, 0.1, 0.5 and 1.0 mM); at concentrations ≥0.1 mM most zygotes fail to progress beyond the 8-cell stage regardless of target, indicating non-specific morpholino toxicity, whereas at 0.01 mM most zygotes reach the blastocyst stage in both groups, with anti-*Zp2* producing a modestly greater developmental impairment than anti-*Gfp*. Panel (B) is a dot plot of ZP2 immunofluorescence integrated density in 8-cell embryos injected with 0.01 mM anti-*Zp2* morpholino versus anti-*Gfp* control morpholino, accompanied by representative micrographs; the anti-*Zp2* morpholino reduced 8-cell-stage ZP2 signal by approximately 20% compared to the control.

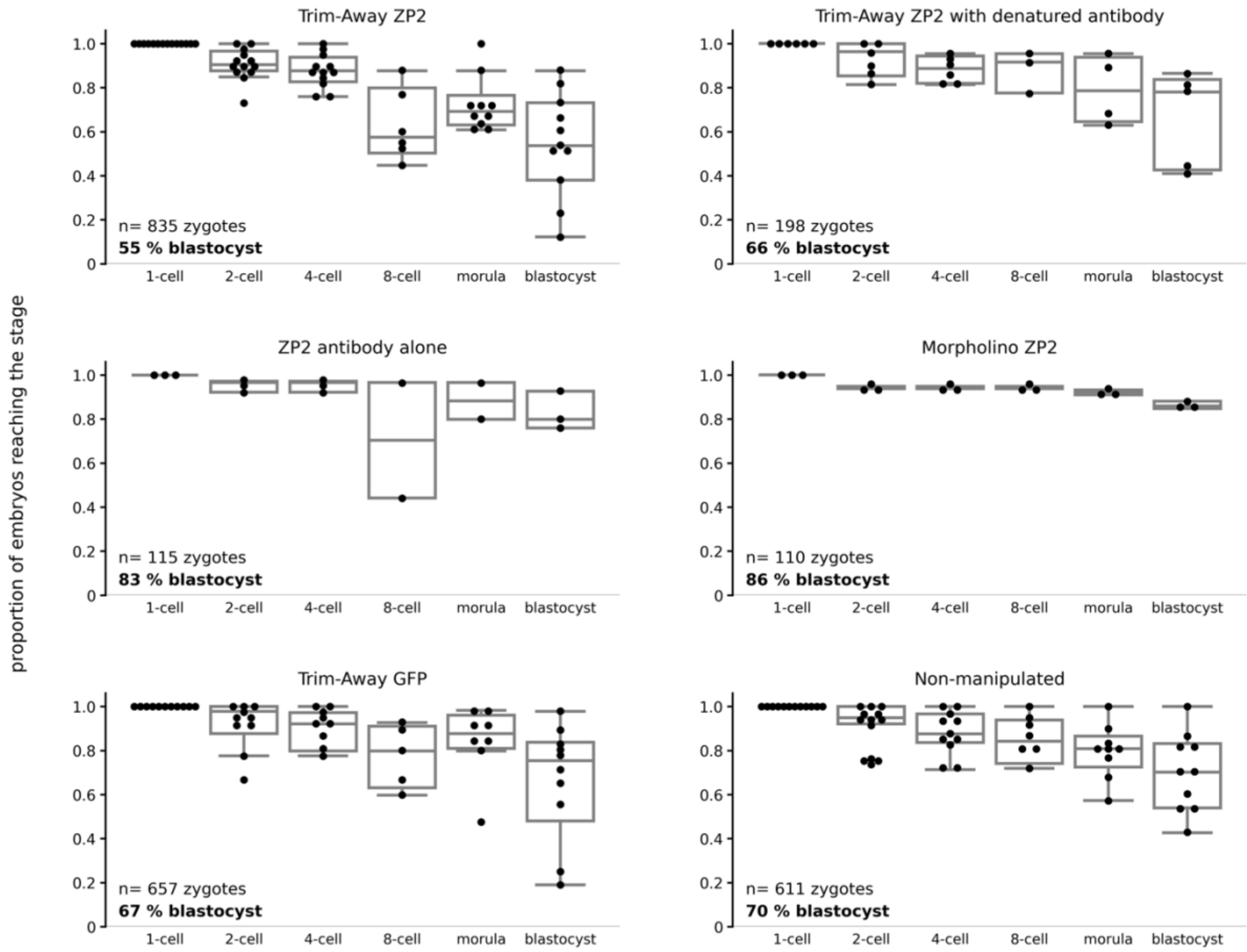

Supplementary Figure S5. Additional treatments as controls for the effects of ZP2 KD, mock KD and MC (Figure 5A). 'Trim-Away ZP2' consisted of zygotes that were microinjected with a cocktail made of *mCherry-Trim21 mRNA* and ZP2 antibody. 'Trim-Away ZP2 with denatured antibody' consisted of zygotes that were microinjected with a cocktail made of *mCherry-Trim21* mRNA and heat-denatured ZP2 antibody. 'ZP2 antibody alone' consisted of zygotes that were microinjected with the sole antibody. 'Morpholino ZP2' consisted of zygotes that were microinjected with the sole morpholino. 'Trim-Away GFP' consisted of zygotes that were microinjected with a cocktail made of *mCherry-Trim21* mRNA and GFP antibody. 'Non-manipulated' consisted of zygotes that were cultured without any treatment. 'n' indicates the total number of starting 1-cell embryos. % blastocysts indicates the average blastocyst rate.

Alt text. Plots showing the proportion of embryos reaching each preimplantation stage, from 1-cell to blastocyst, in six control treatments: Trim-Away ZP2 (n = 835, 55 % blastocyst), Trim-Away ZP2 with denatured antibody (n = 198, 66 %), ZP2 antibody alone (n = 115, 83 %), Morpholino ZP2 (n = 110, 86 %), Trim-Away GFP (n = 657, 67 %) and non-manipulated (n = 611, 70 %). The developmental rate of Trim-Away ZP2 is the one that declined furthest.

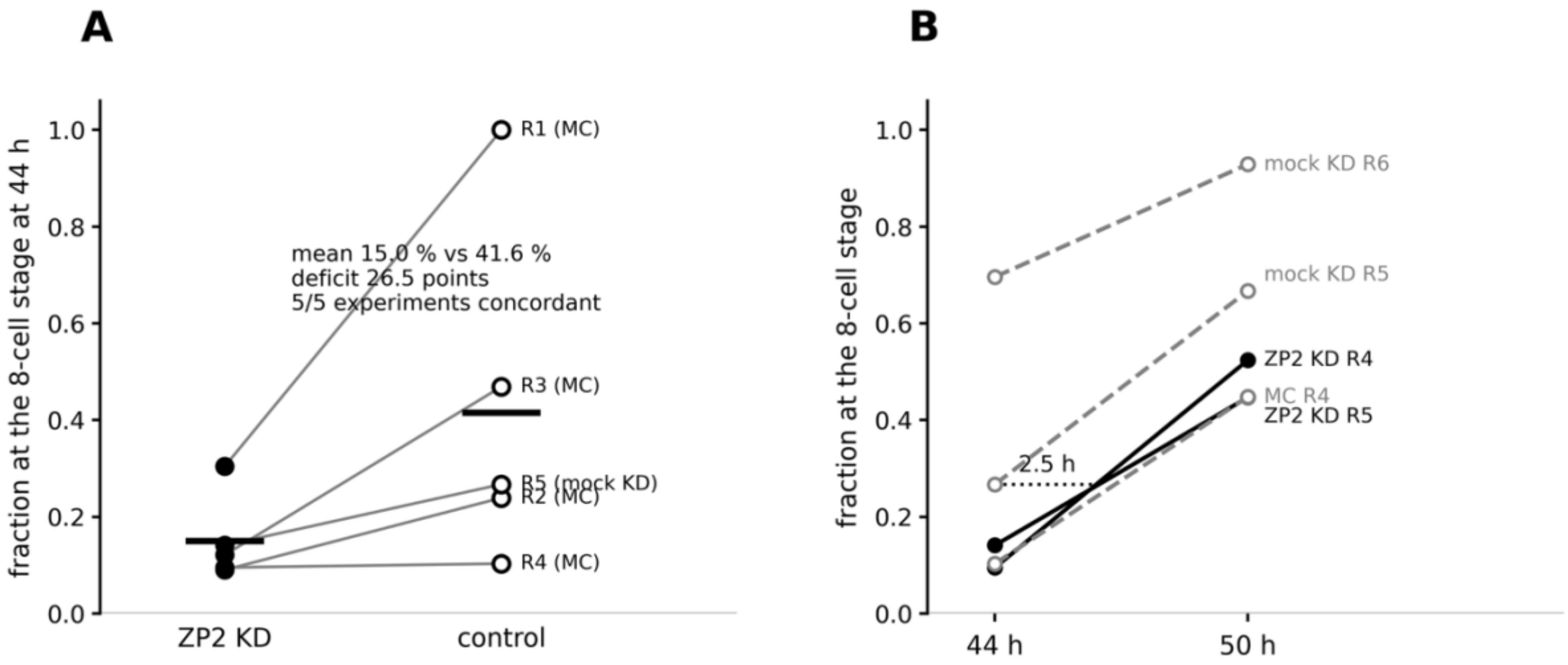

Supplementary Figure S6. Quantification of the developmental delay at the 4-to-8-cell transition after knockdown of ZP2. Embryos were scored at fixed timepoints after completion of the microinjections (1-cell 0 h, 2-cell 20 h, 4-cell 36 h, early 8-cell 44 h, late 8-cell 50 h, morula 72 h, blastocyst 96 h). The 4-to-8-cell transition was chosen because cell number can be scored unambiguously, whereas cavitation changes morphologically within minutes and the cell number of a cavitating embryo cannot be determined without nuclear staining. Replicates are numbered R1-R6. (A) Fraction of embryos that had reached the 8-cell stage at the 44 h scoring, in five replicates (R1-R5) in which ZP2 KD and a control were processed side by side; the control group of each replicate is given in parentheses. Filled symbols, ZP2 KD; open symbols, the paired control; each line joins the two groups of one replicate; horizontal bars are group means. On average 15.0 % of ZP2 KD embryos had reached the 8-cell stage compared with 41.6 % of controls, a deficit of 26.5 percentage points, with all five replicates concordant in direction. (B) Fraction at the 8-cell stage at the 44 h and 50 h scorings, for every replicate in which both were recorded. Solid black lines, ZP2 KD; dashed grey lines, controls. The dotted horizontal line marks the interval by which ZP2 KD embryos lagged in reaching the fraction that the paired control had reached at 44 h. Given a mean arrival rate of 6.1 percentage points per hour between the two scorings, the delay corresponds to approximately 2.5 h. Abbreviations: ZP2 KD, Trim-Away ZP2; mock KD, Trim-Away GFP; MC, micromanipulation control (*mCherry-Trim21* mRNA without antibody).

Alt text. Quantification of how far behind ZP2-depleted embryos were at the 4-to-8-cell transition. Panel (A) is a paired plot with two positions on the x-axis, ZP2 KD and control, and the fraction of embryos at the 8-cell stage at 44 h on the y-axis from 0 to 1. Five lines each join the two groups of one replicate; in every replicate the control point lies above the ZP2 KD point. Horizontal bars mark the group means at 0.150 for ZP2 KD and 0.416 for the controls. Panel (B) plots the same fraction at two timepoints, 44 h and 50 h, for the five replicates in which both scorings were recorded; solid black lines are ZP2 KD and dashed grey lines are controls, and all lines rise between the two timepoints. A dotted horizontal line marks the time interval by which the ZP2 KD curve reaches the level that the paired control had already reached at 44 h, illustrating a lag of about two and a half hours in that experiment.

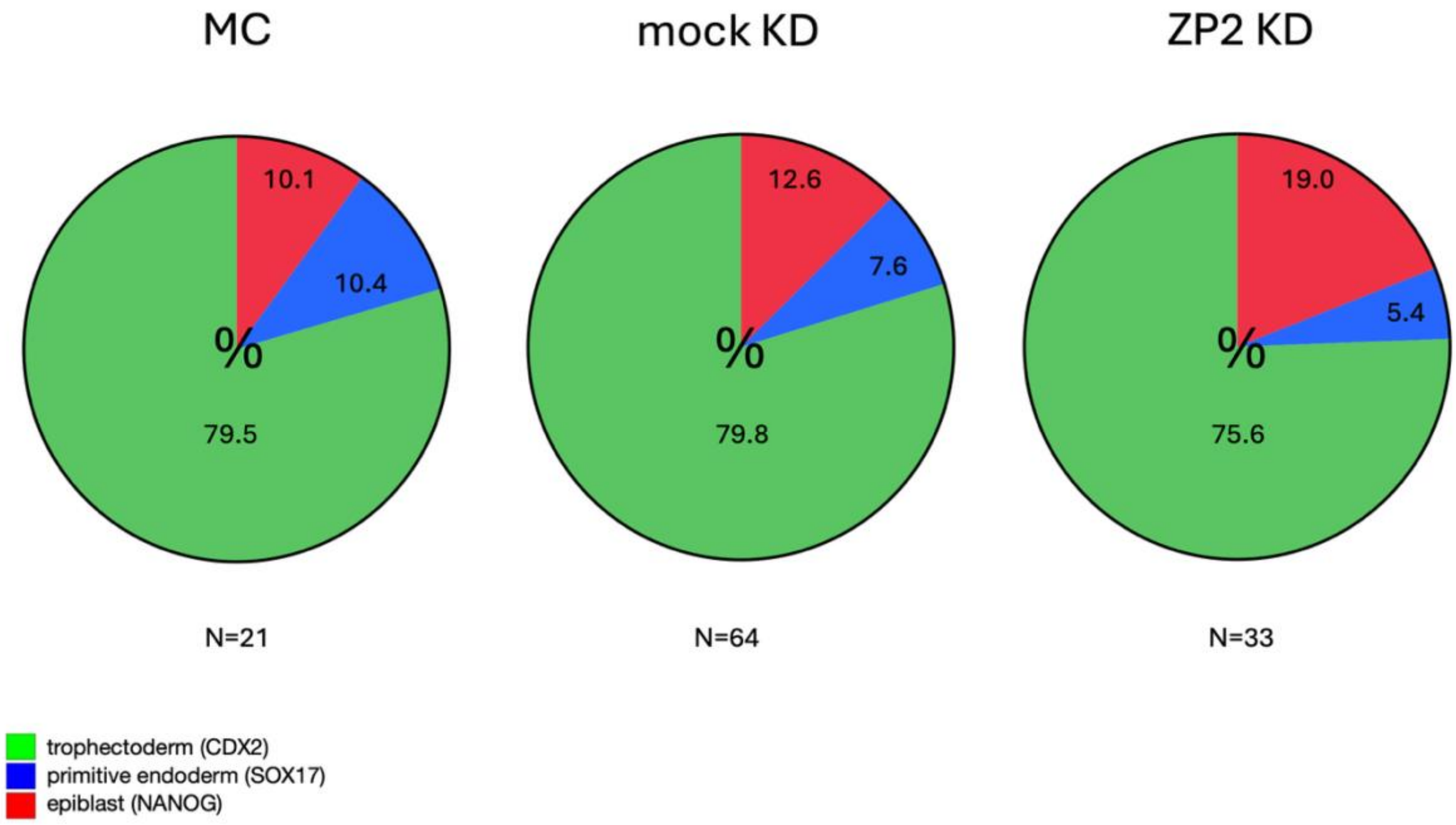

Supplementary Figure S7. Relative proportions of cell lineages in ZP2 KD blastocysts and controls (total cell number set to 100%). At the blastocyst stage (96h post fertilization), ZP2-KD embryos (n=33) were deficient in trophectoderm and primitive endoderm compared to mock KD (n=64) and MC (n=21) embryos. The raw data of the cell counts originated from three experiments and are provided in Supplementary Table S5. Abbreviations: ZP2 KD, ZP2 knockdown by Trim-Away ZP2 + morpholino anti-*Zp2*; mock KD, mock knockdown by Trim-Away GFP + morpholino anti-*Gfp*; MC, micromanipulation control (overexpression of *mCherry-Trim21* mRNA without antibody or morpholino).

Alt text. Pie charts showing the relative proportions (as percentages of total cells) of the three blastocyst cell lineages — CDX2-positive trophectoderm, SOX17-positive primitive endoderm and NANOG-positive epiblast — at 96 h post-fertilization across the three treatment groups (MC n=21, mock KD n=64, ZP2 KD n=33 blastocysts). Compared to MC and mock KD blastocysts, ZP2 KD blastocysts show a reduced relative proportion of trophectoderm and primitive endoderm and a correspondingly increased relative proportion of epiblast.

.

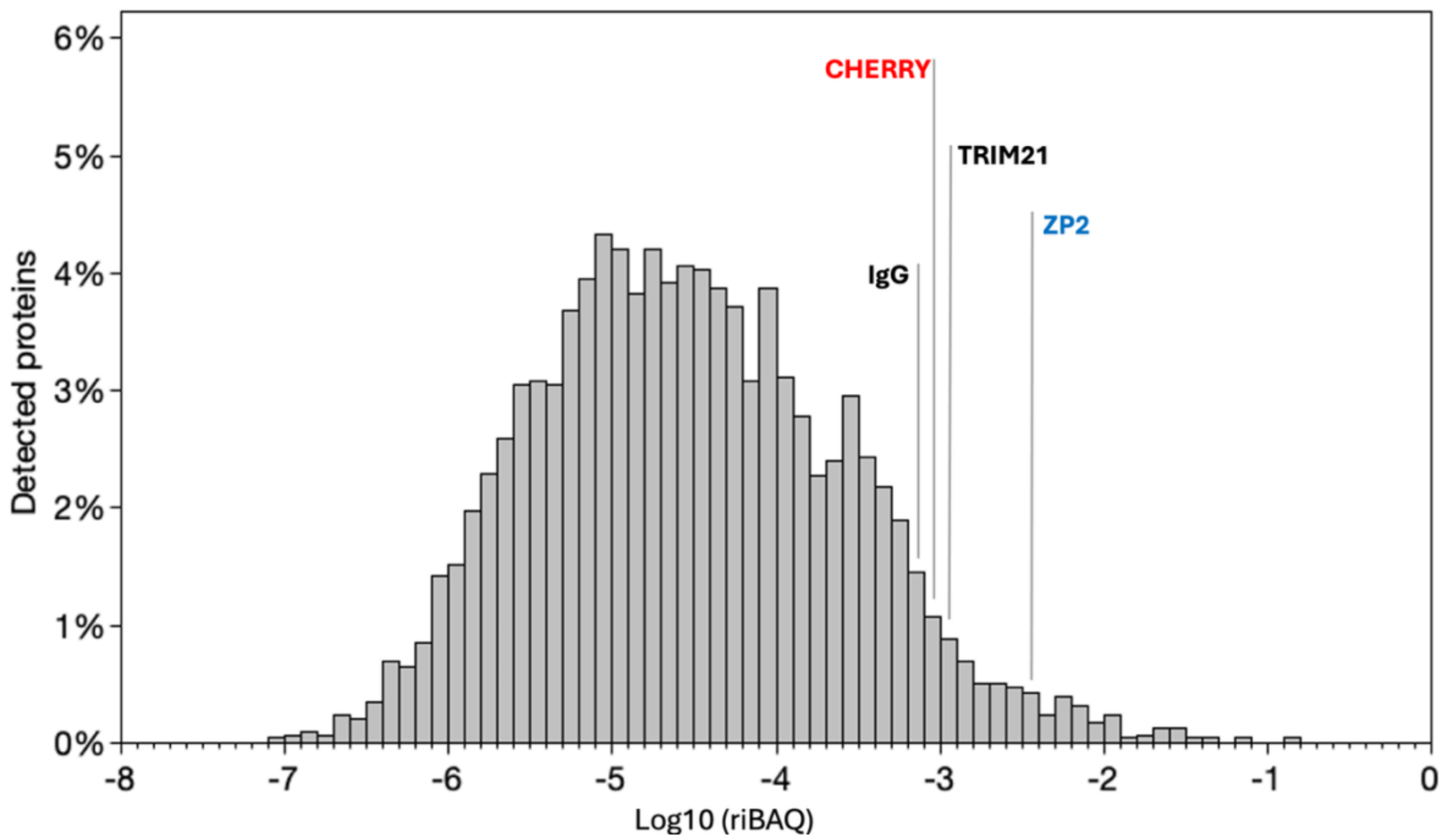

Supplementary Figure S8. ZP2 is present in molar excess over the Trim-Away reagents. Histogram distribution of molar abundances for the proteins detected and quantified by mass spectrometry in ZP2 KD embryos at the 2-cell stage, rendered as % of detected proteins (y-axis) vs $\log_{10}$ riBAQ (x-axis). Zygotes were injected with either antibody (IgG) alone or *mCherry-Trim21* mRNA alone, and analyzed at the 2-cell stage so as to allow the mRNA to accumulate translated product. Vertical lines mark the $\log_{10}$ riBAQ of the substrate ZP2 (blue) and of the three components of the depletion machinery: the microinjected anti-ZP2 antibody (IgG), TRIM21, and the CHERRY (red) tag of the mCherry–TRIM21 reporter. ZP2 lies to the right of - i.e. is more abundant than - the antibody, TRIM21 and CHERRY, indicating molar excess of substrate over machinery. This imbalance is consistent with the partial (≈50%) depletion achieved despite maximal, non-toxic reagent concentrations. Source of dataset: Nolte T, Israel S, Boiani M: Targeted Degradation of Mouse Embryo Proteins Using Trim-Away. In: The Preimplantation Embryo. Edited by Harvey AJ, Gardner DK: Springer Nature; 2026. Abbreviations: IgG, immunoglobulin G; riBAQ, relative intensity-Based Absolute Quantification.

Alt text. Histogram showing the distribution of protein molar abundances, expressed as $\log_{10}$ relative iBAQ (riBAQ) values on the x-axis, for all proteins quantified by mass spectrometry in ZP2 knockdown mouse embryos at the 2-cell stage after microinjection of the individual components of the Trim-Away cocktail at the zygote stage. The bulk of the proteome forms an approximately bell-shaped distribution centred around the lower (more negative) $\log_{10}$ riBAQ values, with progressively fewer proteins toward the higher-abundance (less negative) right-hand tail. Four individual proteins are marked by labelled vertical lines overlaid on the distribution: the substrate ZP2 (blue) and the three components of the Trim-Away depletion machinery, namely the microinjected anti-ZP2 antibody (IgG), TRIM21, and the CHERRY (red) tag of the mCherry–TRIM21 reporter. The IgG, CHERRY and TRIM21 lines cluster together within the high-abundance right tail, while the ZP2 line sits further to the right than all three, indicating that ZP2 is more abundant on a molar basis than the antibody, TRIM21 and CHERRY. The figure therefore shows that the substrate (ZP2) is present in molar excess over the depletion machinery, consistent with the partial (≈50%) depletion achieved despite the use of maximal, non-toxic reagent concentrations.

Supplementary tables

https://doi.org/10.6084/m9.figshare.30405655

Supplementary Table S1: proteomes of zona-intact and zona-free mouse embryos at preimplantation stages, quantified by LC-MS/MS in data-dependent acquisition mode

Supplementary Table S2: normalized ZP2 immunofluorescence intensity values of mouse embryos at preimplantation stages (Dragonfly microscope).

Supplementary Table S3: developmental rates and ZP2 immunofluorescence intensities of mouse embryos after treatment with morpholinos anti-*Zp2* or anti-*Gfp*

Supplementary Table S4: Developmental rates of mouse embryos after ZP2 KD, mock KD, MC and further controls

Supplementary Table S5: Cell lineage composition of blastocysts obtained from mouse embryos subjected to ZP2 KD, mock KD, MC

Supplementary Table S6: Outgrowth formation of blastocysts obtained from mouse embryos subjected to ZP2 KD, mock KD, MC

Supplementary Table S7: Postimplantation development of blastocysts obtained from mouse embryos subjected to ZP2 KD, mock KD, MC

Supplementary Table S8: intensity values of 4531 proteins (all values including zeroes) detected in late morulae of mouse embryos subjected to ZP2 KD, mock KD, MC, measured by LC-MS/MS in data-independent acquisition mode_PXD062983

Supplementary Table S9: intensity values of 2401 proteins (non-zero values) detected in late morulae of mouse embryos subjected to ZP2 KD, mock KD, MC, measured by LC-MS/MS in data-independent acquisition mode_PXD062983

Supplementary Table S10: Venn diagram intersections of differentially expressed transcripts or proteins in ZP2 KD, mock KD, and MC

Supplementary Table S11: FPKM values of 37740 RNAs (all RNA categories) detected in blastocysts of mouse embryos subjected to ZP2 KD, mock KD, MC_E-MTAB-16339

Supplementary Table S12: FPKM values of 17341 RNAs (all RNA categories present in all samples in all replicates, no zero values) detected in blastocysts of mouse embryos subjected to ZP2 KD, mock KD, MC_E-MTAB-16339

Supplementary Table S13: FPKM values of 12920 mRNAs, no zero values, only protein coding, detected in blastocysts of mouse embryos subjected to ZP2 KD, mock KD, MC_E-MTAB-16339

Supplementary Table S14: Ribosomal and stress control gene_sets from Suppl. Tables S9 and S13

References

1. Gao Y, Liu X, Tang B, Li C, Kou Z, Li L, Liu W, Wu Y, Kou X, Li J et al: Protein Expression Landscape of Mouse Embryos during Pre-implantation Development. Cell Rep 2017, 21(13):3957-3969.
2. Rai A, Poh QH, Okae H, Arima T, Totonchi M, Greening DW: Dynamic Proteome Landscape During Preimplantation Human Embryo Development and Trophectoderm Stem Cell-Differentiation. Proteomics 2025, 25(15):72-89.
3. Taher L, Israel S, Drexler HCA, Makalowski W, Suzuki Y, Fuellen G, Boiani M: The proteome, not the transcriptome, predicts that oocyte superovulation affects embryonic phenotypes in mice. Sci Rep 2021, 11(1):23731.
4. Xiong Z, Xu K, Lin Z, Kong F, Wang Q, Quan Y, Sha QQ, Li F, Zou Z, Liu L et al: Ultrasensitive Ribo-seq reveals translational landscapes during mammalian oocyte-to-embryo transition and pre-implantation development. Nat Cell Biol 2022, 24(6):968-980.
5. Zhang H, Ji S, Zhang K, Chen Y, Ming J, Kong F, Wang L, Wang S, Zou Z, Xiong Z et al: Stable maternal proteins underlie distinct transcriptome, translatome, and proteome reprogramming during mouse oocyte-to-embryo transition. Genome Biol 2023, 24(1):166.
6. Zou Z, Zhang C, Wang Q, Hou Z, Xiong Z, Kong F, Wang Q, Song J, Liu B, Liu B et al: Translatome and transcriptome co-profiling reveals a role of TPRXs in human zygotic genome activation. Science 2022, 378(6615):abo7923.
